# Free-energy perturbation in the exchange-correlation space accelerated by machine learning: application to silica polymorphs

**DOI:** 10.1038/s41524-025-01874-1

**Published:** 2025-12-20

**Authors:** Axel Forslund, Jong Hyun Jung, Yuji Ikeda, Blazej Grabowski

**Affiliations:** 1 Institute for Materials Science, University of Stuttgart, Stuttgart, Germany; 2 Department of Materials Science and Engineering, KTH Royal Institute of Technology, Stockholm, Sweden

**Keywords:** Engineering, Materials science, Mathematics and computing, Physics

## Abstract

We propose a free-energy-perturbation approach accelerated by machine-learning potentials to efficiently compute transition temperatures and entropies for all rungs of Jacob’s ladder. We apply the approach to the dynamically stabilized phases of SiO_2_, which are characterized by challengingly small transition entropies. All investigated functionals from rungs 1–4 fail to predict an accurate transition temperature by 25–200%. Only by ascending to the fifth rung, within the random phase approximation, an accurate prediction is possible, giving a relative error of 5%. We provide a clear-cut procedure and relevant data to the community for, e.g., developing and evaluating new functionals.

## Introduction

The search for the ultimate exchange-correlation functional in density functional theory (DFT) is the most significant challenge for first-principles calculations in materials science. The flora of approximations was divided into rungs on Jacob’s ladder by Perdew and Schmidt^1,2^. Starting from the local density and generalized gradient approximation (LDA and GGA), the sophistication increases up to the highest chemical accuracy. At the top resides the random phase approximation (RPA), which considers exact exchange together with an approximate many-body treatment of correlations^3–7^. Being nonlocal, the latter also effectively includes dispersion interactions, while, for lower rungs, semiempirical dispersion corrections may be required. Despite the increased sophistication, the higher-level approximations do not always lead to improved results^8–10^. Evaluation against experiments is thus critical and has, conventionally, been performed for 0-K properties such as lattice constants and cohesive energies^8–21^, see Table 1. In a few cases, low-temperature approximations were used^22^, which, however, can lead to errors at elevated temperatures^23^. Even worse, dynamically unstable phases, such as titanium alloys, high entropy alloys, certain perovskites, and silica, cannot be treated at all.

**Table 1:** Ab initio studies evaluating the performance of the exchange-correlation treatment (mGGA=meta-GGA, hGGA=hybrid GGA; 0 K equilibrium properties: *a*_0_=lattice constant, *B*=bulk modulus, *E*_coh_=cohesion energy, *E*_form_=formation energy; finite temperature (*T*) properties: *T*_q−c_=transition temperature between *β*-quartz and *β*-cristobalite in SiO_2_, ${\bf{\Delta}}$*S*_q−c_=corresponding transition entropy)

					LDA	GGA	mGGA	hGGA	RPA	
Year	Ref.	Properties		Rung →	1	2	3	4	5	No. of solids
2003	^11^	*a*_0_, *B*	0 K		x	x	x			18
2009	^12^	*a*_0_, *E*_coh_	0 K		x	x	x			36
2009	^13^	*a*_0_, *B*	0 K		x	x	x			60
2011	^14^	*a*_0_, *E*_coh/form_	0 K			x		x	x	30
2011	^15^	*a*_0_, *E*_coh_	0 K		x	x	x			20
2013	^8^	*a*_0_, *E*_coh_	0 K			x	x		x	30
2013	^9^	*a*_0_, *B*	0 K		x	x	x	x	x	6
2014	^17^	*E*_coh_	0 K		x	x		x	x	20
2015	^18^	*a*_0_	0 K		x	x	x			20
2016	^10^	*a*_0_, *E*_coh_, *B*	0 K		x	x	x	x		49
2018	^19^	*a*_0_, *E*_form_	0 K			x	x			200
2022	^20^	*a*_0_, *E*_form_	0 K			x	x			6000
2023	^21^	*a*_0_, *E*_form_	0 K			x	x			1000
This work		finite *T*, *T*_q−c_, $\Delta$*S*_q−c_, dynamically stabilized			x	x	x	x	x	SiO_2_ *β*-phases

Silica, SiO_2_, is often used as a model system by virtue of its technologically relevant polymorphs^9,24^. At higher temperatures, the *β*-phases are stable, i.e., *β*-quartz, *β*-cristobalite, and *β*-tridymite (*P*6_3_/*mmc*-tridymite)^25^. These phases are dynamically stabilized and, thus, require explicit finite-temperature modeling. An additional challenge is the stringent requirement on the statistical and computational precision of the Gibbs energy differences when targeting phase transitions. The Gibbs energies of the *β*-phases are similar in magnitude and temperature dependence, so small entropy differences drive the transitions. Even minute inaccuracies of a few meV/atom can shift transition temperatures by several hundred K.

**Fig. 1:**
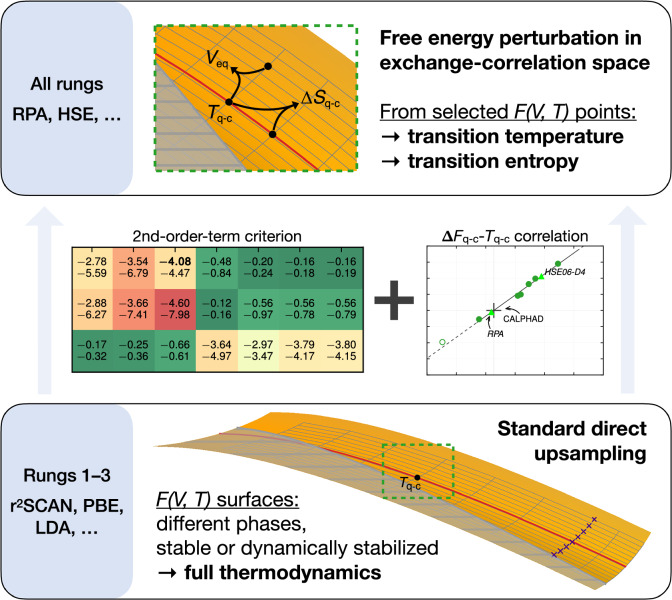
Outline of the developed methodology. Within the lower rungs of Jacob’s ladder of DFT, Helmholtz energy surfaces *F*(*V*, *T*) are computed, yielding the full set of thermodynamic properties (bottom panel). Precise free energy perturbation is then guaranteed by small second-order terms, making all rungs efficiently accessible at selected volume-temperature points (top panel). The correlation with the *β*-quartz–*β*-cristobalite Helmholtz energy difference $\Delta$*F*_q−c_ allows an accurate estimate of the transition temperature *T*_q−c_ and further transition characteristics.

Here, we develop an approach (see Fig. 1) that facilitates the prediction of high-accuracy transition temperatures and entropies for all rungs of Jacob’s ladder. A key component is free-energy perturbation in the exchange-correlation space. The approach works genuinely at finite temperatures, enabling the treatment of dynamically stabilized phases with the same high precision as phases that are stable already at 0 K. We apply the approach to the *β*-phases of silica, focusing on the transition between *β*-quartz and *β*-cristobalite. We compute the transition temperature *T*_q−c_ and transition entropy $\Delta$*S*_q−c_ between these two phases for all rungs of Jacob’s ladder up to the RPA level. Analysis of the results provides a simple geometrical understanding of the phase-space differences between the different exchange-correlation treatments.

## Results

### Direct upsampling for lower rung functionals

**Fig. 2:**
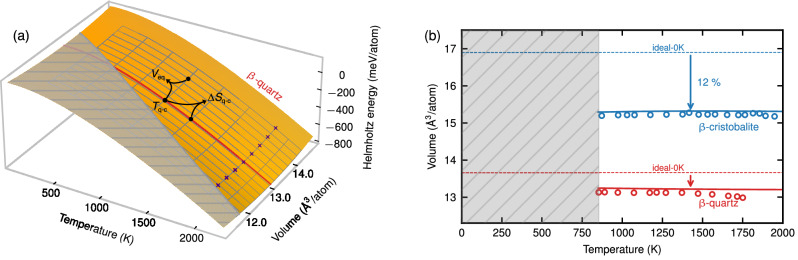
Calculated Helmholtz energy and volume within selected exchange-correlation functionals. **a** Helmholtz energy surface for *β*-quartz within LDA. The red line marks the volume expansion at ambient pressure. The black dots indicate volume-temperature points for the calculation of the transition temperature, *T*_q−c_, transition entropy, $\Delta$*S*_q−c_, and equilibrium volume, *V*_eq_, for rungs 4 and 5 within the proposed approach. The purple crosses indicate the points used for training the machine-learning potential. **b** Volume expansion at ambient pressure of *β*-quartz and *β*-cristobalite within r^2^SCAN-D4. Finite-temperature results (solid lines) are compared with experimental data (circles)^56–61^ and results for symmetry-constrained idealized structures at 0 K (dashed lines). The gray areas mark the dynamical instability regime. See Supplementary Fig. S1 for volume-expansion and bulk-modulus results for various rung 1–3 functionals.

The proposed approach is based on the direct upsampling method^26^. Within the latter, machine-learning potentials (MLPs) are utilized to efficiently compute a high-precision Helmholtz energy surface with well-converged computational parameters (e.g., a dense volume-temperature mesh and supercells with several thousand atoms), isolating the inherent error due to the exchange-correlation approximation and enabling its evaluation. Direct upsampling can be applied well for rungs 1–3 from Jacob’s ladder. For the present case, we compute Helmholtz energy surfaces for the silica *β*-phases utilizing various functionals from rungs 1–3 (LDA^27^, GGA-PBE^28^, meta-GGA r^2^SCAN^29^ with and without dispersion corrections). The *β*-quartz LDA surface is shown in Fig. 2a as an example. The gray-shaded region marks the instability regime where the system transforms into *α*-quartz, highlighting the necessity of an explicit finite-temperature treatment. Various thermodynamic quantities can be extracted from the Helmholtz energy surface, e.g., the thermal expansion, as emphasized by the red line. Figure 2b compares the volume expansion for *β*-quartz and *β*-cristobalite within r^2^SCAN, including the D4 correction, with experiments marked by circles. The explicit finite-temperature results (solid lines) give a good prediction, while calculations for the symmetry-constrained idealized 0-K structures are meaningless (dashed lines). A comparison of various functionals from rungs 1–3 with experiments is provided in Supplementary Sec. S2. LDA shows a good agreement for the volume at *T*_q−c_ with a deviation below 1.5%, but fails to correctly predict the temperature dependence. The third rung meta-GGA functionals, including dispersion corrections, give the best results with deviations below 1%, qualitatively reproducing the temperature dependence.

**Fig. 3:**
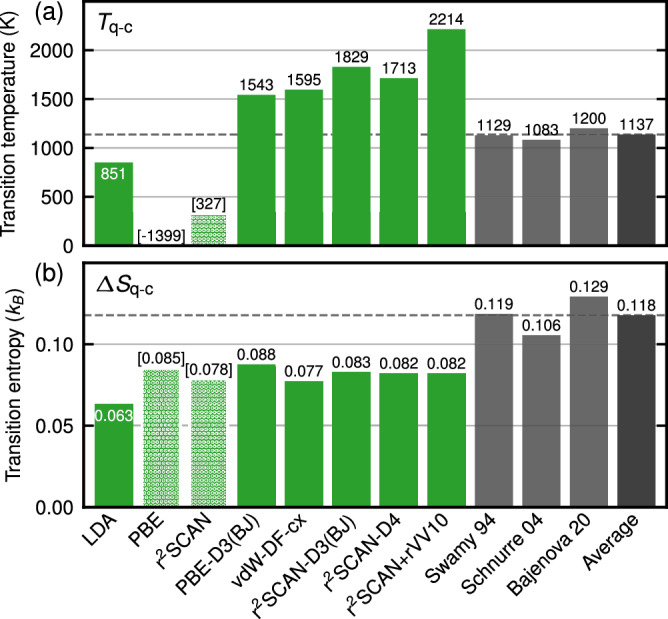
*β*-quartz to *β*-cristobalite transition properties. **a** Transition temperatures *T*_q−c_ and **b** Transition entropies $\Delta$*S*_q−c_ in SiO_2_ for rungs 1–3 functionals (green) compared with CALPHAD^25,62,63^ (gray). Extrapolated temperatures are indicated with brackets, and the CALPHAD average is marked with a dashed line.

The crossing of the *β*-quartz and *β*-cristobalite Gibbs energies determines *T*_q−c_. The Gibbs energies are obtained by a Legendre transformation of the Helmholtz energy surfaces. The resulting *T*_q−c_’s from rungs 1–3 are compared in Fig. 3a with CALPHAD values representing experiments. The CALPHAD values vary marginally and we take the average for comparison. The spread of the ab initio predicted values is, on the other hand, significant. Without dispersion corrections, *T*_q−c_ is underestimated. LDA’s *T*_q−c_ is 251 K (22%) below CALPHAD, while PBE’s and r^2^SCAN’s predictions even fall out of the stability window of the phases. Including dispersion corrections shifts *T*_q−c_ upward, resulting in overestimations, e.g., 408 K (36%) for PBE-D3(BJ) or 594 K (52%) for r^2^SCAN-D4. The corresponding transition entropies $\Delta$*S*_q−c_ (differences between the slopes of the Gibbs energies at *T*_q−c_) are shown in Fig. 3b. All rung 1–3 functionals underestimate $\Delta$*S*_q−c_ with the largest discrepancy of 56% for LDA.

### Systematic approach for higher-rung predictions

In light of the discrepancies in predicting *T*_q−c_ and $\Delta$*S*_q−c_ observed for the functionals of rungs 1–3, an extension of the analysis to higher rungs is desirable. The difficulty is that the computational requirements for rungs 4 and 5 increase strongly. In the following, we describe a systematically extendable approach that overcomes these difficulties and enables—in successive steps—an accurate prediction of the transition temperature and entropy for rungs 4 and 5.

The approach builds on two main observations extracted from the wide range of results obtained for rungs 1–3. (1) The transition temperature *T*_q−c_ is well correlated with $\Delta$*F*_q−c_, the Helmholtz energy difference between the two phases for a certain, specified condition. (2) Free energy perturbation can be utilized to efficiently obtain Helmholtz energy differences between the functionals. Using these observations, it is possible to drastically reduce the computational effort in predicting *T*_q−c_ and $\Delta$*S*_q−c_ within rungs 4 and 5. Observation (1) allows us to focus on a single volume-temperature point for each of the two phases for the *T*_q−c_ prediction (cf. Fig. 2a for *β*-quartz), reducing the number of calculations by about two orders of magnitude. The entropy $\Delta$*S*_q−c_ can be obtained by one additional point on each surface. Observation (2) dispenses with the necessity to fit MLPs for rungs 4 or 5. Instead, the optimized potentials from rungs 1–3 are used to generate suitable snapshots. Taken together, the prediction of *T*_q−c_ and $\Delta$*S*_q−c_ for rungs 4 or 5 can be obtained in a few hundred snapshots of supercells with about two hundred atoms.

**Fig. 4:**
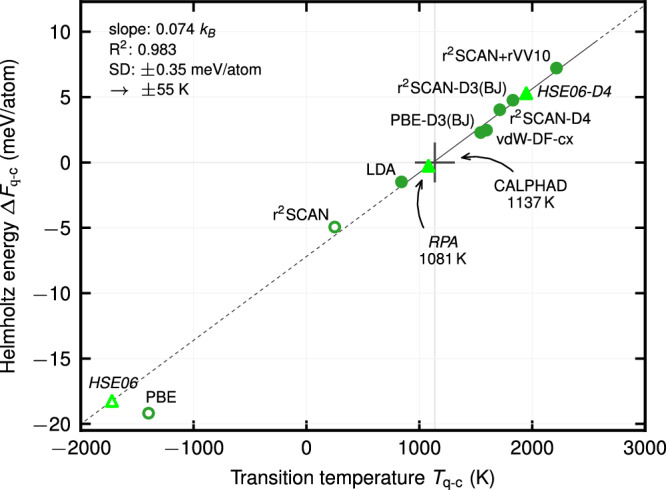
Correlation between the predicted transition temperature and Helmholtz energy difference between *β*-quartz and *β*-cristobalite at ${T}_{{\rm{q}}-{\rm{c}}}^{\exp }$, and ${V}_{{\rm{cristobalite}}}^{\exp }$ and ${V}_{{\rm{quartz}}}^{\exp }$. The filled dark green circles show explicitly computed results and the line a linear fit of them. The hollow circles mark extrapolations out of the stability regime of the phases. The light green triangles mark predictions for rung 4 and 5 functionals (labeled in italics) utilizing the fit.

The correlation between *T*_q−c_ and $\Delta$*F*_q−c_ is displayed in Fig. 4. The difference $\Delta$*F*_q−c_ (*y*-axis) is obtained as

1 $$\Delta {F}_{{\rm{q}}-{\rm{c}}}={F}_{{\rm{cristobalite}}}({V}_{{\rm{cristobalite}}}^{\exp },{T}_{{\rm{q}}-{\rm{c}}}^{\exp })-{F}_{{\rm{quartz}}}({V}_{{\rm{quartz}}}^{\exp },{T}_{{\rm{q}}-{\rm{c}}}^{\exp }),$$

where *F*_cristobalite_ and *F*_quartz_ correspond to the Helmholtz energies for a given exchange-correlation functional; further, ${T}_{{\rm{q}}-{\rm{c}}}^{\exp }$ is the experimental (or CALPHAD) transition temperature, and ${V}_{{\rm{cristobalite}}}^{\exp }$ and ${V}_{{\rm{quartz}}}^{\exp }$ are the experimental equilibrium volumes of the two phases at ${T}_{{\rm{q}}-{\rm{c}}}^{\exp }$. The *T*_q−c_ value (*x*-axis) corresponds to the self-consistently computed transition temperature of the respective functional. The such obtained (*T*_q−c_, $\Delta$*F*_q−c_)-pairs are shown by the dark green dots in Fig. 4. The good linear relation is quantified by the small standard deviation of the fit of 0.35 meV/atom, which propagates into an uncertainty of 55 K in the transition temperature prediction. Even the predictions from r^2^SCAN and PBE, which fall outside the stability regime of the phases, are reasonably captured by extrapolation. This extrapolative capacity is useful for locating functionals with low predictability of *T*_q−c_, e.g., HSE06 (see Fig. 4). However, for the main purpose of locating functionals with high predictability, we rely only on the interpolative behavior of the linear relation. The best prediction is quantified by a vanishing $\Delta$*F*_q−c_, which corresponds to the averaged CALPHAD transition temperature of 1137 K.

To obtain the transition entropy $\Delta$*S*_q−c_, we utilize a finite difference,

2 $$\Delta {S}_{{\rm{q}}-{\rm{c}}}=(\Delta {F}_{{\rm{q}}-{\rm{c}}}-\Delta {F}_{{\rm{q}}-{\rm{c}}}^{{\prime} })/\Delta T,$$

where $\Delta {F}_{{\rm{q}}-{\rm{c}}}^{{\prime} }$ is a second Helmholtz energy difference computed similarly as $\Delta$*F*_q−c_ in Eq. (1) but at a shifted temperature. The linear temperature dependence of the free energy difference between the two phases (see Suplementary Figs. S4, S5, S7) allows us to utilize a large enough temperature shift $\Delta$*T* (≈300 K) to guarantee a numerically stable evaluation of $\Delta$*S*_q−c_.

To compute the Helmholtz energy for each of the phases in Eq. (1) for functionals of rung 4 or 5, we utilize free-energy perturbation in the exchange-correlation space (second observation). Specifically, at volume *V* and temperature *T*,

3 $$F(V,T)={F}^{\rm{ML}}(V,T;{\rm{xc}}^{1-3})+\Delta {F}^{\rm{up}}(V,T;{\rm{xc}}^{1-3}).$$

Here, *F*^ML^(*V*, *T*; xc^1–3^) is the Helmholtz energy obtained within direct upsampling with an MLP fitted to an exchange-correlation (xc) functional from rung 1–3 and

4 $$\Delta {F}^{{\rm{up}}}(V,T;{{\rm{xc}}}^{{{1-3}}})=-{k}_{B}T\,{\rm{ln}}{\left\langle \exp \left(-\frac{\Delta E}{{k}_{B}T}\right)\right\rangle }_{{\rm{ML}}},$$

where $\Delta$*E* = *E* − *E*^ML^, with the energies *E* and *E*^ML^ calculated with a rung 4 or 5 functional and the xc^1–3^ MLP, respectively. The xc^1–3^ MLP is also used to sample the thermodynamic average in Eq. (4).

### Two classes of functionals

**Fig. 5:**
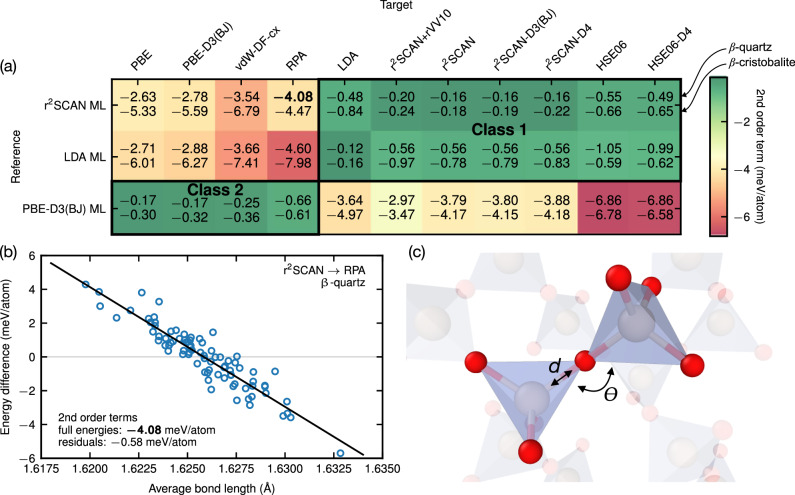
Upsampling performance between functionals. **a** Second-order terms for *β*-quartz / *β*-cristobalite, in meV/atom. The fields are colored according to the average value and qualitatively indicate the resemblance between the phase spaces of the reference and target functionals. **b** Correlation between average Si–O bond length and the energy difference between r^2^SCAN and RPA for *β*-quartz (blue circles). The black line shows a linear fit. Second-order terms from free-energy perturbation using full energy differences and residuals are written in the lower left corner. The values on the *y*-axis are offset with respect to the average energy. **c** Illustration of the Si–O bond length *d* and Si–O–Si bond angle *θ* in silica.

In practice, the convergence behavior of the thermodynamic average with the number of snapshots is decisive. To quantify the convergence behavior, we make use of the second-order terms in the expansion of Eq. (4) (see Supplementary Sec. S3D). Generally, values less than 1 meV/atom allow for efficient use of Eq. (4) (around 100 snapshots for high convergence). Values for various combinations of functionals are given in the matrix in Fig. 5a. Two classes of functionals can be distinguished as highlighted by the two green areas identifying small second-order terms and thus a good overlap between the corresponding functionals in phase space. The first, larger class is composed of LDA, meta-GGA (r^2^SCAN), and the hybrid functional HSE06 with or without dispersion corrections. The second class consists of the GGA functionals (PBE with or without correction), vdW-DF-cx (classified as a nonlocal van der Waals functional^30^), and RPA.

Interclass combinations of functionals show larger second-order terms (yellow to red areas in the matrix). The larger second-order terms can be understood by simple geometrical means. In Fig. 5b, we plot, as an example, the energy differences between r^2^SCAN (represented by the MLP) and RPA as a function of the average Si–O bond length (Fig. 5c). The correlation is good and, in particular, removing the spread in energies due to the bond-length dependence, in practice by subtracting the linear fit, leads to a second-order term of below 1 meV/atom (from −4.08 to −0.58 meV/atom). Si–O–Si bond angles are similarly well correlated as bond lengths—however, the combined account for bond lengths and angles does not improve the correlation, which shows that these two parameters are strongly correlated. The importance of bond lengths and angles has been realized since the early days of the development of classical potentials for SiO_2_^31^. Here, we unveil how even fine nuances in the description of these coordinates (changes in the range of 0.01Å) affect the free-energy perturbation.

### Transition properties for rung 4 and 5

We utilize the proposed approach to compute *T*_q−c_ and $\Delta$*S*_q−c_ for HSE06, HSE06-D4 (both rung 4), and RPA (rung 5) by upsampling, respectively, from r^2^SCAN for the first two and from PBE-D3(BJ) for RPA, chosen according to the smallest corresponding second-order term. The results are included in Fig. 4 by the bright green triangles. For the hybrid-GGA HSE06 functional, the extrapolated transition temperature lies far off, close to that of PBE. The D4 corrections strongly shift the transition temperature upward, resulting in an overestimation. The transition entropy is 0.060 *k*_*B*_ and 0.084 *k*_*B*_, respectively. Overall, no improvement is seen compared to the rung 1–3 functionals. For RPA, our analysis (Supplementary Sec. S3C) shows that extremely well-converged computational settings are required, in particular for the plane wave cutoff (1000 eV), to obtain convergence. We also find that core polarization is important, shifting *T*_q−c_ up by 267 K. The final RPA transition temperature and entropy are 1081 K and 0.088 *k*_*B*_ (underestimation of 5% and 25%), thus being closest to experiment among the investigated exchange-correlation treatments.

## Discussion

The proposed approach is systematically extendable and—based on the provided data^32^ and clear-cut procedure, Supplementary Sec. S1 —straightforwardly available to the community. For example, in developing new functionals, *T*_q−c_ can be used as the first benchmark quantity. The set of dedicated snapshots (100 for each phase) along with the MLP energies for the various rung 1–3 functionals is available for download^32^. To evaluate a new functional, the energies for the snapshots are computed (with any independent code) and utilized in the free-energy perturbation formulas. The predictive capability of the functional can also be tested for the transition entropy by utilizing another set of snapshots. Further, we provide a third set of snapshots with which the equilibrium volume of the new functional at *T*_q−c_ can be predicted (cf. Fig. 2a and Secs. S1C, S2D). Eventually, at a larger computational cost, the full Helmholtz energy surface can be upsampled in the spirit of the direct-upsampling approach, giving access to various thermodynamic quantities (e.g., heat capacity, bulk modulus).

The observed and utilized linearity between *T*_q−c_ and $\Delta$*F*_q−c_ is closely related to the linear temperature dependence of $\Delta$*F*_q−c_ (Supplementary Figs. S4, S5, S7). A linear temperature dependence of free-energy differences between phases is well-known for many unary systems^33^. Within our approach, the validity of the linear assumption increases when the predictions of the evaluated exchange-correlation functional are close to experiment. Should an exchange-correlation functional yield a transition temperature strongly deviated from the experimental value, the assumption of linearity may be invalidated. A similar consideration applies to the fixed-volume assumption, which may be invalidated if the equilibrium volumes of the functional deviate strongly from experiment. In such cases, systematically adding further Helmholtz energy (*V*, *T*) points into the upsampling (as just mentioned above) relaxes the need for the linearity assumption.

We emphasize that the precision required for a meaningful comparison of the *β*-quartz–*β*-cristobalite transition exceeds the one typically achievable with MLPs (see, e.g., Supplementary Fig. S8). Therefore, even with MLPs trained on higher-rung data, inclusion of the free-energy perturbation term $\Delta$*F*^up^ in Eq. (3) remains essential to correct for the intrinsic errors of the MLPs.

The investigated SiO_2_ system entails various challenges (dynamical instability, small transition entropies, soft phonon modes^34^). The successful application of the introduced approach to SiO_2_ indicates that other material systems should be likewise treatable, thus providing a useful tool for exchange-correlation functional development and evaluation at finite temperatures.

One interesting future direction could be the combination of the presented approach with other high-accuracy methods such as CCSD(T)^35^, which achieve chemical accuracy of approximately 1 kcal/mol for a wide range of systems and are therefore regarded as the gold standard in computational chemistry. A practical implementation could be achieved together with the recently developed local CCSD(T) methods for periodic systems^36,37^ and with MLPs trained on CCSD(T) data^38^.

## Methods

We computed densely sampled Helmholtz energy surfaces for 32 combinations of SiO_2_ phases and exchange-correlation functionals. Specifically, the following four phases were modeled:

1. *β*-quartz^39^
2. *β*-cristobalite^40,41^
3. *P*6_3_/*mmc*-tridymite^42^
4. *C*222_1_-tridymite^43^

For each phase, eight different exchange-correlation functionals from rungs 1–3 of Jacob’s ladder, without and with dispersion corrections, were used:

$$\begin{array}{ll}\begin{array}{ll}1.\ {\rm{LDA}}\\ 2.\ {\rm{PBE}}\\ 3.\ {\rm{r}^{{\rm{2}}}SCAN}\end{array}\\\left.\begin{array}{ll}4.\ {\rm{PBE}\hbox{-}D3(BJ)}\\ 5.\ {\rm{vdW\hbox{-}DF\hbox{-}cx}}\\ 6.\ {\rm{r}^{{\rm{2}}}SCAN\hbox{-}D3(BJ)}\\ 7.\ {\rm{r}^{{\rm{2}}}SCAN\hbox{-}D4}\\ 8.\ {\rm{r}^{{\rm{2}}}SCAN\hbox{+}rVV10}\end{array}\quad\right\}\,{\rm{dispersion}}\ {\rm{corrected}}\end{array}$$

(References and abbreviation definitions are given below.) The full direct upsampling procedure was used to calculate Helmholtz energy surfaces for LDA, r^2^SCAN and PBE-D3(BJ). For all other functionals, we used the free-energy-perturbation step between exchange-correlation functionals. For rungs 1–3, we computed the full Helmholtz energy surfaces, while for the higher-rung HSE06 and RPA we used our developed procedure with a few selected volume-temperature points, as summarized in Fig. 1 and described in the Results [Eqs. (1)–(4) and related text].

### Direct upsampling

The direct upsampling was performed in the extended version of ref. ^44^, due to the dynamical instability at 0 K of the investigated phases. In particular, effective equations of state were used as the basis for the finite-temperature calculations. These equations of state were adjusted to allow for reasonable thermodynamic integration. Further, an effective harmonic reference fitted to high temperatures was used as a reference for thermodynamic integration to a moment tensor potential (MTP)^45^, specifically trained for each phase. To ensure convergence with respect to supercell size, the thermodynamic integrations were performed with up to around six thousand atoms. All supercell sizes and the parameters of the effective equations of state, harmonic references and MTPs are detailed in Supplementary Sec. S3A.

The DFT calculations were performed with the projector-augmented wave (PAW) method^46,47^ as implemented in the Vienna ab initio simulation package (VASP)^48^. PBE-based PAW potentials were used for all but the LDA calculations, for which LDA-based potentials were applied. A valence-electron configuration of Si: 3*s*^2^3*p*^2^; O: 2*s*^2^2*p*^4^ was used. For the RPA calculations, the VASP _GW PBE PAW potentials were used, as well as an additional PAW potential for the Si core-polarization correction, see Supplementary Sec. S3C. The utilized functionals from rungs 1–3 follow:

- LDA, local density approximation^27^
- GGA-PBE, generalized gradient approximation parametrized by Perdew, Burke, Ernzerhof^28^
- r^2^SCAN, regularized-restored strongly constrained and appropriately normed meta-GGA functional^29^
- vdW-DF-cx, nonlocal van der Waals density functional with consistent exchange^30^ GGA-PBE-D3(BJ)^49^
- r^2^SCAN-D3(BJ)^49,50^
- r^2^SCAN-D4^50^
- r^2^SCAN+rVV10^51^

where

- D3(BJ), van der Waals interaction of Grimme with Becke–Johnson damping^49^
- D4, van der Waals interaction of Grimme^52^
- rVV10, revised nonlocal van der Waals density functional^53^

From rungs 4 and 5, we used:

- HSE06, Heyd-Scuseria-Ernzerhof hybrid functional^54^
- HSE06-D4^52,55^
- RPA, random phase approximation within the adiabatic connection fluctuation-dissipation theorem^3,4^

Detailed DFT parameters and convergence checks are presented in Supplementary Secs. S3A and S3B.

### Random phase approximation

Due to the significant computational requirements of RPA calculations, a special procedure was devised to obtain the RPA results. To compute the RPA upsampling energy $\Delta$*F*^up^, around 50 snapshots are needed to provide a good statistical convergence. The size of the supercell for the upsampling is around 200 atoms, specifically 243 atoms for *β*-quartz and 192 atoms for *β*-cristobalite. In order for such calculations to be computationally feasible, we compute, in the first step of the procedure, $\Delta {F}_{{\rm{normal}}}^{{\rm{up}}}$ with a set of normally converged parameters. The requirement on these parameters is to provide converged second-order and higher-order terms of the free-energy perturbation. In the second step, we add a first-order correction calculated with a smaller snapshot (9/24 atoms for *β*-quartz/*β*-cristobalite). Using a smaller snapshot allows us to use highly converged computational parameters that ensure the highest precision, also with respect to the relative stability between the phases. This first-order correction is strictly valid when the energy of the smaller snapshot is converged with respect to the snapshot average energy. The exact parameters used, resulting intermediate energies and detailed convergence tests can be found in Supplementary Sec. S3C. Ultimately, the upsampling Helmholtz energy is obtained as

5 $$\Delta {F}^{{\rm{up}}}=\Delta {F}_{{\rm{normal}}}^{{\rm{up}}}+{E}_{{\rm{high}}}-{E}_{{\rm{normal}}},$$

where *E*_high_ is the highly converged energy per atom of the small snapshot, and *E*_normal_ is the energy per atom of the small snapshot scaled to 243 or 192 atoms and with identical parameters to the upsampling.

## Supplementary information

Supplementary Information

## Data Availability

The data needed for reproducing the results presented in this paper are available on Darus at 10.18419/DARUS-4999 (ref. ^32^).

## Code availability

The code for Direct Upsampling is available at https://gitlab.com/axefor/direct-upsampling. Scripts for the developed procedure are available at 10.18419/DARUS-4999 (ref. ^32^).

## References

[1] Perdew, J. P. Jacob’s ladder of density functional approximations for the exchange-correlation energy. In *AIP Conference Proceedings* Vol. 577, 1–20 (AIP, Antwerp (Belgium), 2001).

[2] Perdew, J. P. et al. Prescription for the design and selection of density functional approximations: more constraint satisfaction with fewer fits. *J. Chem. Phys.* **123**, 062201 (2005). 10.1063/1.1904565 16122287

[3] Langreth, D. C. & Perdew, J. P. Exchange-correlation energy of a metallic surface: Wave-vector analysis. *Phys. Rev. B* **15**, 2884–2901 (1977).

[4] Kaltak, M., Klimeš, J. & Kresse, G. Cubic scaling algorithm for the random phase approximation: self-interstitials and vacancies in Si. *Phys. Rev. B* **90**, 054115 (2014).

[5] Ren, X., Rinke, P., Joas, C. & Scheffler, M. Random-phase approximation and its applications in computational chemistry and materials science. *J. Mater. Sci.* **47**, 7447–7471 (2012).

[6] Hellgren, M. & Baguet, L. Random phase approximation with exchange for an accurate description of crystalline polymorphism. *Phys. Rev. Res.* **3**, 033263 (2021).

[7] Sengupta, N., Bates, J. E. & Ruzsinszky, A. From semilocal density functionals to random phase approximation renormalized perturbation theory: a methodological assessment of structural phase transitions. *Phys. Rev. B* **97**, 235136 (2018).

[8] Schimka, L., Gaudoin, R., Klimeš, J., Marsman, M. & Kresse, G. Lattice constants and cohesive energies of alkali, alkaline-earth, and transition metals: random phase approximation and density functional theory results. *Phys. Rev. B* **87**, 214102 (2013).

[9] Xiao, B. et al. Testing density functionals for structural phase transitions of solids under pressure: Si, SiO_2_, and Zr. *Phys. Rev. B* **88**, 184103 (2013).

[10] Tran, F., Stelzl, J. & Blaha, P. Rungs 1 to 4 of DFT Jacob’s ladder: extensive test on the lattice constant, bulk modulus, and cohesive energy of solids. *J. Chem. Phys.* **144**, 204120 (2016). 27250292 10.1063/1.4948636

[11] Tao, J., Perdew, J. P., Staroverov, V. N. & Scuseria, G. E. Climbing the density functional ladder: nonempirical meta–generalized gradient approximation designed for molecules and solids. *Phys. Rev. Lett.* **91**, 146401 (2003). 14611541 10.1103/PhysRevLett.91.146401

[12] Perdew, J. P., Ruzsinszky, A., Csonka, G. I., Constantin, L. A. & Sun, J. Workhorse semilocal density functional for condensed matter physics and quantum chemistry. *Phys. Rev. Lett.* **103**, 026403 (2009). 19659225 10.1103/PhysRevLett.103.026403

[13] Haas, P., Tran, F. & Blaha, P. Calculation of the lattice constant of solids with semilocal functionals. *Phys. Rev. B* **79**, 085104 (2009).

[14] Schimka, L., Harl, J. & Kresse, G. Improved hybrid functional for solids: the HSEsol functional. *J. Chem. Phys.* **134**, 024116 (2011). 21241089 10.1063/1.3524336

[15] Sun, J. et al. Self-consistent meta-generalized gradient approximation within the projector-augmented-wave method. *Phys. Rev. B* **84**, 035117 (2011).

[16] Cohen, A. J., Mori-Sánchez, P. & Yang, W. Challenges for density functional theory. *Chem. Rev.* **112**, 289–320 (2012). 22191548 10.1021/cr200107z

[17] Olsen, T. & Thygesen, K. S. Accurate ground-state energies of solids and molecules from time-dependent density-functional theory. *Phys. Rev. Lett.* **112**, 203001 (2014).

[18] Sun, J., Ruzsinszky, A. & Perdew, J. P. Strongly constrained and appropriately normed semilocal density functional. *Phys. Rev. Lett.* **115**, 036402 (2015). 26230809 10.1103/PhysRevLett.115.036402

[19] Zhang, Y. et al. Efficient first-principles prediction of solid stability: towards chemical accuracy. *npj Comput. Mater.* **4**, 9 (2018).

[20] Kingsbury, R. et al. Performance comparison of *r*^2^SCAN and SCAN metaGGA density functionals for solid materials via an automated, high-throughput computational workflow. *Phys. Rev. Mater.* **6**, 013801 (2022).

[21] Kothakonda, M. et al. Testing the r^2^SCAN density functional for the thermodynamic stability of solids with and without a van der Waals correction. *ACS Mater. Au* **3**, 102–111 (2023). 38089726 10.1021/acsmaterialsau.2c00059 PMC9999476

[22] Wei, J., Xia, Z., Xia, Y. & He, J. Hierarchy of exchange-correlation functionals in computing lattice thermal conductivities of rocksalt and zinc-blende semiconductors. *Phys. Rev. B* **110**, 035205 (2024).

[23] Grabowski, B. et al. Ab initio vibrational free energies including anharmonicity for multicomponent alloys. *npj Comput. Mater.* **5**, 80 (2019).

[24] Demuth, T., Jeanvoine, Y., Hafner, J. & Ángyán, J. G. Polymorphism in silica studied in the local density and generalized-gradient approximations. *J. Condens. Matter Phys.* **11**, 3833–3874 (1999).

[25] Schnurre, S., Gröbner, J. & Schmid-Fetzer, R. Thermodynamics and phase stability in the Si–O system. *J. Non-Cryst. Solids* **336**, 1–25 (2004).

[26] Jung, J. H., Srinivasan, P., Forslund, A. & Grabowski, B. High-accuracy thermodynamic properties to the melting point from ab initio calculations aided by machine-learning potentials. *npj Comput. Mater.* **9**, 3 (2023).

[27] Ceperley, D. M. & Alder, B. J. Ground state of the electron gas by a stochastic method. *Phys. Rev. Lett.* **45**, 566–569 (1980).

[28] Perdew, J. P., Burke, K. & Ernzerhof, M. Generalized gradient approximation made simple. *Phys. Rev. Lett.* **77**, 3865–3868 (1996). 10062328 10.1103/PhysRevLett.77.3865

[29] Furness, J. W., Kaplan, A. D., Ning, J., Perdew, J. P. & Sun, J. Accurate and numerically efficient r^2^SCAN meta-generalized gradient approximation. *J. Phys. Chem. Lett.* **11**, 8208–8215 (2020). 32876454 10.1021/acs.jpclett.0c02405

[30] Berland, K. & Hyldgaard, P. Exchange functional that tests the robustness of the plasmon description of the van der Waals density functional. *Phys. Rev. B* **89**, 035412 (2014).

[31] Vashishta, P., Kalia, R. K., Rino, J. P. & Ebbsjö, I. Interaction potential for SiO_2_: a molecular-dynamics study of structural correlations. *Phys. Rev. B* **41**, 12197–12209 (1990). 10.1103/physrevb.41.12197 9993674

[32] Forslund, A., Jung, J. H., Ikeda, Y. & Grabowski, B. *Data for: Free-energy perturbation in the exchange-correlation space accelerated by machine learning: application to silica polymorphs DaRUS*, V1 10.18419/DARUS-4999 (2025). 10.1038/s41524-025-01874-1 PMC12783050 41522821

[33] Dinsdale, A. SGTE data for pure elements. *Calphad* **15**, 317–425 (1991).

[34] Tezuka, Y., Shin, S. & Ishigame, M. Observation of the silent soft phonon in *β*-quartz by means of hyper-raman scattering. *Phys. Rev. Lett.* **66**, 2356–2359 (1991). 10043464 10.1103/PhysRevLett.66.2356

[35] Raghavachari, K., Trucks, G. W., Pople, J. A. & Head-Gordon, M. A fifth-order perturbation comparison of electron correlation theories. *Chem. Phys. Lett.* **157**, 479–483 (1989).

[36] Ye, H.-Z. & Berkelbach, T. C. Adsorption and vibrational spectroscopy of CO on the surface of MgO from periodic local coupled-cluster theory. *Faraday Discuss.* **254**, 628–640 (2024). 39049598 10.1039/d4fd00041b PMC11539119

[37] Ye, H.-Z. & Berkelbach, T. C. Periodic local coupled-cluster theory for insulators and metals. *J. Chem. Theory Comput.* **20**, 8948–8959 (2024). 39376105 10.1021/acs.jctc.4c00936

[38] Ikeda, Y. et al. *Machine-learning interatomic potentials achieving CCSD(T) accuracy for systems with extended covalent networks and van der Waals interactions* http://arxiv.org/abs/2508.14306, ArXiv:2508.14306 [cond-mat] (2025). 10.1021/acs.jctc.5c02045 PMC13019697 41774831

[39] Bragg, W. L. & Gibbs, R. E. The structure of *α* and *β* quartz. *Proc. R. Soc. Lond. A* **109**, 405–427 (1925).

[40] Wyckoff, R. W. G. Crystal structure of high temperature cristobalite. *Am. J. Sci.* **s5-9**, 448–459 (1925).

[41] Wyckoff, R. W. G. IX. Die Kristallstruktur von *β*-Cristobalit SiO_2_ (bei hohen Temperaturen stabile Form). *Z. Kristallogr. Cryst. Mater.* **62**, 189–200 (1925).

[42] Kihara, K. Thermal change in unit-cell dimensions, and a hexagonal structure of tridymite. *Z. Kristallogr. Cryst. Mater.* **148**, 237–254 (1978).

[43] Dollase, W. A. The crystal structure at 220^∘^C of orthorhombic high tridymite from the Steinbach meteorite. *Acta Crystallogr.* **23**, 617–623 (1967).

[44] Jung, J. H., Forslund, A., Srinivasan, P. & Grabowski, B. Dynamically stabilized phases with full ab initio accuracy: thermodynamics of Ti, Zr, Hf with a focus on the hcp-bcc transition. *Phys. Rev. B* **108**, 184107 (2023).

[45] Shapeev, A. V. Moment tensor potentials: a class of systematically improvable interatomic potentials. *Multiscale Model Simul.* **14**, 1153–1173 (2016).

[46] Blöchl, P. E. Projector augmented-wave method. *Phys. Rev. B* **50**, 17953–17979 (1994). 10.1103/physrevb.50.17953 9976227

[47] Kresse, G. & Joubert, D. From ultrasoft pseudopotentials to the projector augmented-wave method. *Phys. Rev. B* **59**, 1758–1775 (1999).

[48] Kresse, G. & Furthmüller, J. Efficient iterative schemes for ab initio total-energy calculations using a plane-wave basis set. *Phys. Rev. B* **54**, 11169–11186 (1996). 10.1103/physrevb.54.11169 9984901

[49] Grimme, S., Ehrlich, S. & Goerigk, L. Effect of the damping function in dispersion corrected density functional theory. *J. Comput. Chem.* **32**, 1456–1465 (2011). 21370243 10.1002/jcc.21759

[50] Ehlert, S. et al. r^2^SCAN-D4: dispersion corrected meta-generalized gradient approximation for general chemical applications. *J. Chem. Phys.* **154**, 061101 (2021). 33588552 10.1063/5.0041008

[51] Ning, J. et al. Workhorse minimally empirical dispersion-corrected density functional with tests for weakly bound systems: r^2^SCAN + rVV10. *Phys. Rev. B* **106**, 075422 (2022).

[52] Caldeweyher, E., Mewes, J.-M., Ehlert, S. & Grimme, S. Extension and evaluation of the D4 London-dispersion model for periodic systems. *Phys. Chem. Chem. Phys.* **22**, 8499–8512 (2020). 32292979 10.1039/d0cp00502a

[53] Sabatini, R., Gorni, T. & de Gironcoli, S. Nonlocal van der Waals density functional made simple and efficient. *Phys. Rev. B* **87**, 041108 (2013).

[54] Krukau, A. V., Vydrov, O. A., Izmaylov, A. F. & Scuseria, G. E. Influence of the exchange screening parameter on the performance of screened hybrid functionals. *J. Chem. Phys.* **125**, 224106 (2006). 17176133 10.1063/1.2404663

[55] Grimme, S. *Generally Applicable Atomic-Charge Dependent London Dispersion Correction DFTD4* https://github.com/dftd4/dftd4 (2025). 10.1063/1.5090222 31005066

[56] Berger, C., Eyrand, L., Richard, M. & Rivière, R. Etude radiocristallographique de variation de volume pour quelques matériaux subissant des transformation des phases solide-solide. *Bull. Soc. Chim. Fr.* **2**, 628–633 (1966).

[57] Ackermann, R. J. & Sorrell, C. A. Thermal expansion and the high–low transformation in quartz. I. High-temperature X-ray studies. *J. Appl. Crystallogr.* **7**, 461–467 (1974).

[58] Wright, A. F. & Leadbetter, A. J. The structures of the *β*-cristobalite phases of SiO_2_ and AlPO_4_. *Philos. Mag.* **31**, 1391–1401 (1975).

[59] Ohsumi, K., Sawada, T., Takeuchi, Y. & Sadanaga, R. *Laser-Heating Device for Single-Crystal Diffractometry and Its Application to the Structural Study of High Cristobalite*. Materials science of the Earth’s interior (D. Reidel, 1984).

[60] Swainson, I. P. & Dove, M. T. On the thermal expansion of *β*-cristobalite. *Phys. Chem. Miner.* **22**, 61–65 (1995).

[61] Bourova, E. & Richet, P. Quartz and cristobalite: high-temperature cell parameters and volumes of fusion. *Geophys. Res. Lett.* **25**, 2333–2336 (1998).

[62] Swamy, V., Saxena, S. K., Sundman, B. & Zhang, J. A thermodynamic assessment of silica phase diagram. *J. Geophys. Res.Solid Earth* **99**, 11787–11794 (1994).

[63] Bajenova, I., Khvan, A., Dinsdale, A. & Kondratiev, A. Implementation of the extended Einstein and two-state liquid models for thermodynamic description of pure SiO_2_ at 1 atm. *Calphad* **68**, 101716 (2020).

