# Supplementary material for: Free-energy perturbation in the exchange-correlation space accelerated by machine learning: application to silica polymorphs
Source: NPJ Comput Mater. 2025 Dec 20;12(1):14. doi: 10.1038/s41524-025-01874-1 (PMC12783050; doi:10.1038/s41524-025-01874-1)
Supplement: Supplementary file 1 — Supplementary Information [file 41524_2025_1874_MOESM1_ESM.pdf]

Supplementary Information to: Free-energy perturbation in the exchange-correlation space accelerated by machine learning: Application to silica polymorphs

Axel Forslund[1,2,*] Jong Hyun Jung[1], Yuji Ikeda[1], and Blazej Grabowski[1]
[1]Institute for Materials Science, University of Stuttgart, Pfaffenwaldring 55, 70569 Stuttgart and
[2]Department of Materials Science and Engineering, KTH Royal Institute of Technology, SE-100 44 Stockholm, Sweden
(Dated: November 6, 2025)

CONTENTS

S1. Procedure for functional evaluation 2
A. Step-by-step instructions for $T_{\text{q-c}}$ 2
B. Transition entropy 2
C. Equilibrium volumes 2
D. Procedure using the DIRECT UPSAMPLING `python` package 2

S2. Results and Discussion 3
A. Finite temperature properties and comparison to experiments 3
B. Phase stabilities 3
C. Quartz-cristobalite transition 3
D. Equilibrium volume estimates 5

S3. Methods 8
A. Detailed computational parameters 8
B. Systematic tests for convergence and accuracy 12
C. RPA details and convergence tests 14
D. Expansion of free-energy perturbation 14

Fig. S1: T-dependent volume expansion and bulk modulus
Fig. S2: Volume at 1137 K referenced to experiment
Fig. S3: Absolute volume and bulk modulus at 1137 K
Fig. S4: Gibbs energy differences (phase stabilities)
Fig. S5: Shifted Gibbs energy difference
Fig. S6: Correlation $T_{\text{q-c}} - \Delta G_{\text{q-c}}$ and $T_{\text{q-c}} - \Delta F_{\text{q-c}}$
Fig. S7: Calculation of $\Delta S_{\text{q-c}}$ for HSE06(-D4)
Fig. S8: RMSEs for high-MTP training
Fig. S9: Supercell-size convergence for F^{ah}
Fig. S10: Cutoff convergence for r^{2}SCAN upsampling
Fig. S11: Basis impact in F^{ah} description
Fig. S12: Mesh impact in F^{ah} description
Fig. S13: RPA energies vs. time/frequency points
Fig. S14: RPA k-point convergence
Fig. S15: RPA cutoff-energy convergence

Table S1: Rung 1–3 transition properties
Table S2: Rung 4 and 5 transition properties
Table S3: HSE06 and HSE06-D4 equilibrium volumes
Table S4: Methodological overview
Table S5: Supercells for direct upsampling
Table S6: Parameters for low-DFT calculations
Table S7: Snapshot generation with low-MTP
Table S8: Low-MTP fitting and results
Table S9: High-MTP fitting and results
Table S10: Effective harmonic potentials
Table S11: High-DFT parameters
Table S12: Parameters for TI and FEP
Table S13: Basis for $F^{\text{ah}}(V,T)$
Table S14: Upsampling for RPA
Table S15: RPA first-order correction
Table S16: Final RPA results
Table S17: Parameters for RPA calculations

S1. PROCEDURE FOR FUNCTIONAL EVALUATION

The transition temperature, $T_{\text{q-c}}$, and the transition entropy, $\Delta S_{\text{q-c}}$, between β-quartz and β-cristobalite can be estimated for any new functional and/or DFT setup using the approach established in the main text, together with snapshots provided online [1] (in `input_functional_evaluation`).

A. Step-by-step instructions for $T_{\text{q-c}}$

Step 1: Snapshots for the $T_{\text{q-c}}$ calculation are downloaded (subfolder `a.transition_temperature`; file format: VASP `POSCAR`). They are uncorrelated (separated by enough steps) and have been pre-generated for both phases (`quartz_beta`, `cristobalite_beta`) with three machine-learning (ML) potentials (`lda_mtp`, `pbe_d3bj_mtp`, `r2scan_mtp`).

Step 2: The energies of the snapshots are calculated by the user with the new functional and/or DFT setup.

Step 3: The second-order term (Sec. S3 D) is determined for all potentials and both phases as

$$e_2 = -\frac{1}{2k_B T_{\text{q-c}}^{\text{exp}}}\left[\frac{1}{N_{\text{s}}}\sum_{i=1}^{N_{\text{s}}}\Delta E_i^2 - \left(\frac{1}{N_{\text{s}}}\sum_{i=1}^{N_{\text{s}}}\Delta E_i\right)^2\right], \tag{S1}$$

with $T_{\text{q-c}}^{\text{exp}} = 1137\,\text{K}$ and $\Delta E_i = E_i - E_i^{\text{ML}}$, the difference between the user-calculated and ML-potential energy for the i'th snapshot with N_{a} atoms. The sums run over the provided $N_{\text{s}} = 100$ snapshots for each phase. The ML energies, E_i^{ML}, are provided in the file `energy_mtp` in the snapshot folder. The potential giving the smallest e_2's is selected for the next steps. The e_2's should be $\lesssim 2$ meV/atom to guarantee convergence within the number of provided snapshots. Larger values indicate the inability to predict $T_{\text{q-c}}$.

Step 4: The Helmholtz energy difference between the new functional/DFT setup and the selected ML potential is determined with free-energy perturbation. In particular, for each phase (p = cristobalite or quartz),

$$\Delta F_p^{\text{up}} = -\frac{k_B T_{\text{q-c}}^{\text{exp}}}{N_{\text{a}}}\ln\left[\frac{1}{N_{\text{s}}}\sum_{i=1}^{N_{\text{s}}}\exp\left(-\frac{\Delta E_i}{k_B T_{\text{q-c}}^{\text{exp}}}\right)\right]. \tag{S2}$$

Step 5: The Helmholtz energy difference between β-quartz and β-cristobalite is calculated by

$$\Delta F_{\text{q-c}} = \Delta F_{\text{cristobalite}}^{\text{up}} - \Delta F_{\text{quartz}}^{\text{up}} + \Delta F_{\text{q-c}}^{\text{ML}}, \tag{S3}$$

where the first two terms are given by Eq. (S2), and $\Delta F_{\text{q-c}}^{\text{ML}}$ is the Helmholtz energy difference on the level of the ML potential, evaluated in this work and given as (1137 K-column for $T_{\text{q-c}}$; unit: meV/atom):

	1137 K	1419 K
LDA MTP:	−1.34	−2.84
PBE-D3(BJ) MTP:	+2.07	+0.12
r^{2}SCAN MTP:	−5.30	−6.90

Step 6: Finally, the transition temperature is calculated by

$$T_{\text{q-c}} \approx T_{\text{q-c}}^{\text{exp}} + \frac{\Delta F_{\text{q-c}}}{0.074\,k_B} \tag{S4}$$

and compared with the CALPHAD average value $T_{\text{q-c}}^{\text{exp}} = 1137$ K. Equation (S4) follows from the relation of $T_{\text{q-c}}$ with the Helmholtz energy difference $\Delta F_{\text{q-c}}$ between the two phases, as established in the main text (see also Sec. S2 C). The value $0.074\,k_B$ corresponds to the slope of this relation.

B. Transition entropy

The transition entropy, $\Delta S_{\text{q-c}}$, can be computed by repeating steps 1, 2, 4, 5 for a new set of snapshots (subfolder `b.transition_entropy`) for the selected ML potential. These snapshots correspond to $T = 1419$ K, which should be used in Eq. (S2). Also, the 1419 K-column should be used in Eq. (S3). The second Helmholtz energy difference $\Delta F'_{\text{q-c}}$ obtained in this way is used together with $\Delta F_{\text{q-c}}$ (i.e., the difference obtained during the $T_{\text{q-c}}$ calculation) in Eq. (2) of the main text with $\Delta T = 282$ K to obtain the transition entropy.

C. Equilibrium volumes

The equilibrium volumes of β-quartz and β-cristobalite can be likewise estimated for the new functional/DFT setup by repeating steps 1, 2, and 4 for a third set of snapshots (subfolder `c.equilibrium_volume`). These snapshots correspond to the experimental transition temperature as for the $T_{\text{q-c}}$ calculation, but are located at different volumes. Using these snapshots in Eq. (S2) gives new $\Delta F_p^{\text{up}\prime\prime}$ values which, together with the original ΔF_p^{up} values from the $T_{\text{q-c}}$ prediction, give correction slopes,

$$v_p = (\Delta F_p^{\text{up}\prime\prime} - \Delta F_p^{\text{up}})/\Delta V, \tag{S5}$$

where $\Delta V = 0.8\,\text{Å}^3$/atom. The linear corrections given by the v_p's need to be added to the original volume-dependent Helmholtz energies for the selected ML potentials for each phase (the offsets do not affect the equilibrium volume and are not relevant). The original Helmholtz energies are stored in text format in the sub-subfolder `helmholtz_energies`. The equilibrium volumes of the new functional/DFT setup can be obtained by finding the minima of the new curves.

D. Procedure using the DIRECT UPSAMPLING `python` package

The procedure of preparing input files and post-processing (steps 2–6) is automated using scripts provided in Ref. [1]. The scripts utilize the DIRECT UPSAMPLING `python` package, available through https://gitlab.com/axefor/direct-upsampling), which requires the Atomic Simulation Environment (ASE) `python` library [2] for input file generation. This allows any compatible calculator class to be used, for example, that of VASP as in the provided example.

S2. RESULTS AND DISCUSSION

In total, 32 densely sampled Helmholtz energy surfaces were obtained. In addition, following the approach proposed in the main text, three volume-temperature (V,T) points were computed with the rung-4 hybrid functional HSE06 with and without the D4 dispersion correction for both the β-quartz and β-cristobalite phases, in order to predict $T_{\text{q-c}}$, $\Delta S_{\text{q-c}}$, and the equilibrium volume of each phase. For the rung-5 random phase approximation (RPA), two (V,T) points were computed for each of the two phases to predict $T_{\text{q-c}}$ and $\Delta S_{\text{q-c}}$.

In the following, we show data complementary to the main text, including temperature dependencies of the equilibrium volumes and bulk moduli, as well as phase stabilities, for the rung-1–3 functionals. We also provide tables that include the data plotted in the main text for the β-quartz–β-cristobalite transition, as well as some additional results obtained with the developed approach.

A. Finite temperature properties and comparison to experiments

Complementary to Fig. 2(b), we show in Fig. S1(a) and (c) detailed results for the volume expansion of β-quartz and β-cristobalite for all the functionals in rungs 1–3. Additionally, in Fig. S1(b) and (d), we show the corresponding adiabatic bulk moduli. The results are compared with experimental data, where available.

A further comparison with experiments, with the temperature fixed to 1137 K, is shown in Fig. S2. The PBE functional significantly overestimates the volume for all phases, while LDA performs quite well. Interestingly, the van der Waals corrected PBE functional, PBE-D3(BJ), gives a similar volume as r^{2}SCAN, while r^{2}SCAN with a van der Waals correction gives the best results. The difference between different types of van der Waals corrections is small in this case (whether local or nonlocal), although r^{2}SCAN-D3(BJ) seems to give the smallest discrepancy with experiments in terms of volume.

We also show bar plots for the absolute volume and the adiabatic bulk modulus at the same temperature of 1137 K in Fig. S3, including additionally $C222_1$-tridymite. The relation between the volumes of the different phases is consistently predicted by all functionals. Turning to the bulk moduli, β-quartz is predicted to be the hardest, consistent with previous predictions [10]. In terms of bulk modulus, the r^{2}SCAN functional, with or without dispersion corrections, gives the results closest to experiments where available (only for β-quartz).

B. Phase stabilities

Figure S4 shows the stability of β-cristobalite and the two tridymite phases with respect to β-quartz at ambient pressure. The Gibbs energy differences between the phases are below 10 meV/atom over almost the whole investigated temperature range for most functionals. Only for the PBE functional, there is an overall larger difference, destabilizing β-quartz with respect to the other phases. This prediction, that β-quartz would be metastable, stands in sharp contrast to the experimental phase diagram. The r^{2}SCAN functional produces qualitatively similar incorrect results as PBE, but with smaller energy differences.

The introduction of van der Waals corrections stabilizes β-quartz in all cases. The relative stability between the two tridymites and β-cristobalite is less affected. This is correlated with the difference in density between the phases: because quartz is denser than the other phases, it gets a stronger stabilizing contribution from the dispersion interactions. The tridymites and β-cristobalite, on the other hand, have densities very similar to each other.

The $P6_3/mmc$-tridymite and β-cristobalite phases have energies that are extremely close to each other, on a level smaller than the precision of the present calculations. It is therefore not possible in the present work to distinguish when or if there is a transition in pure SiO_2. In previous work [12–15], however, it has been suggested that $P6_3/mmc$-tridymite is not formed in pure SiO_2, but only stabilized by impurities. The reason for this could be kinetics or a small energy difference that is not resolvable by DFT calculations. In the present study, we rely on the previous assessments [12, 13] and focus on the β-quartz–β-cristobalite transition, which is well and consistently described within CALPHAD.

We note, though, that all the rung-1–3 functionals predict that the $P6_3/mmc$-tridymite phase is more stable than the $C222_1$-tridymite phase within the investigated temperature range. This is consistent with experiments [16], which have suggested a transition temperature of 693 K.

C. Quartz-cristobalite transition

Transition properties between β-quartz and β-cristobalite derived from the Gibbs energies of the rung-1–3 functionals (Fig. S4) are shown in Table S1. To estimate transition properties for functionals of rungs 4 and 5, we utilize the observed similarity of the $\Delta G(T)$ dependencies for the lower rungs. To see this similarity of the $\Delta G(T)$'s between β-cristobalite and β-quartz clearly, we plot *shifted* Gibbs energy differences in Fig. S5. The shifts have been adjusted to make all curves cross at 1137 K, i.e., at the experimental transition temperature. All investigated rung 1–3 functionals show very linear temperature dependencies with similar slopes. The corresponding transition entropies differ by only a few 0.01 k_B.

Because of the similar $\Delta G(T)$'s between β-cristobalite and β-quartz, the $T_{\text{q-c}}$'s obtained for the different functionals are well correlated with corresponding Gibbs energy differences. We plot this correlation for the functionals of rungs 1–3 in Fig. S6(a). The difference in Gibbs energy is calculated at 1137 K and ambient pressure by optimizing the volumes. For comparison, the correlation of the $T_{\text{q-c}}$'s with the respec-

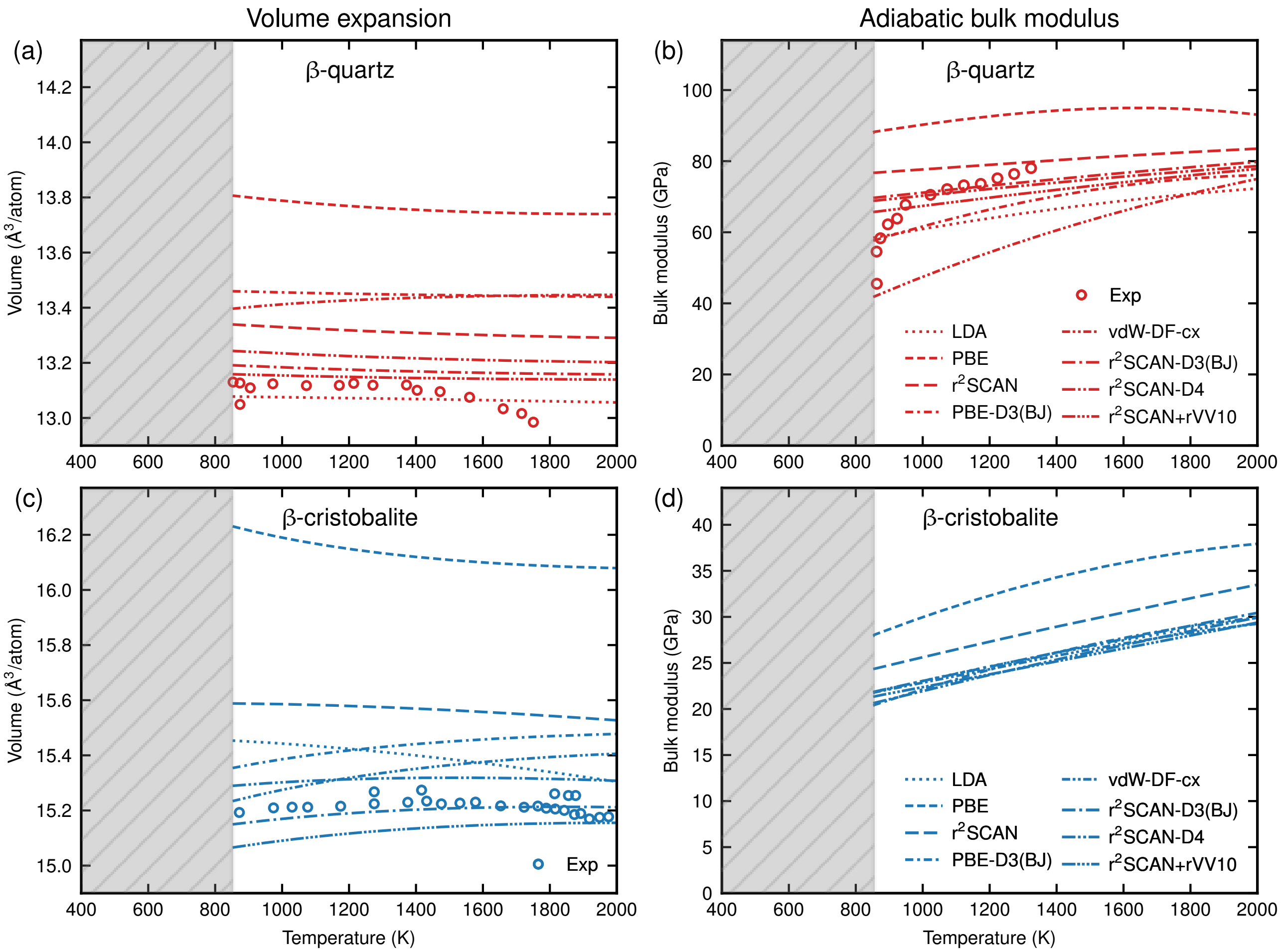

FIG. S1. Rung-1–3 finite-temperature volume [(a) and (c)] and adiabatic bulk modulus [(b) and (d)], for β-quartz [(a) and (b)] and β-cristobalite [(c) and (d)]. The experimental data (circles) are taken from the following references: β-quartz [3–5], β-cristobalite [4, 6, 7], and Refs. [8, 9] as assessed in Ref. [4].

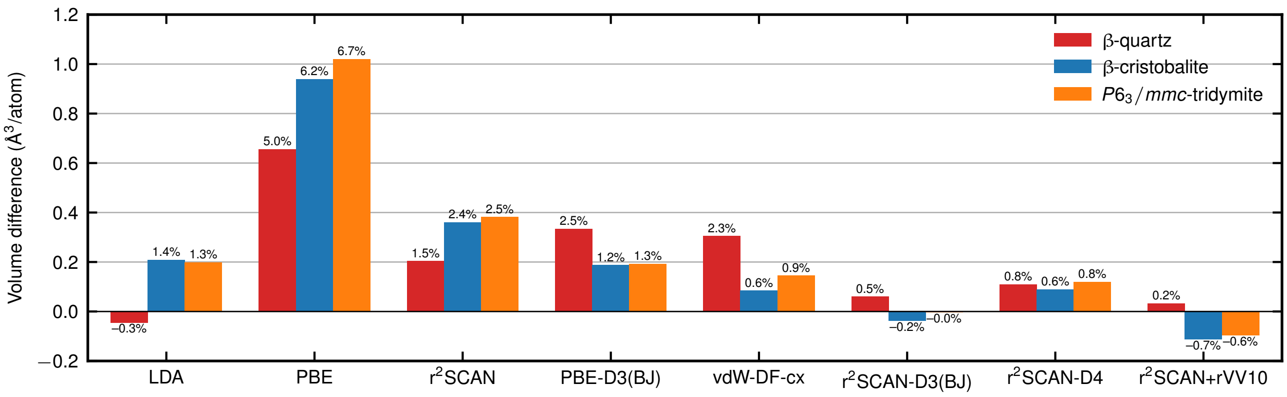

FIG. S2. Rung-1–3 volume difference with respect to experiments [3, 4] at 1137 K of β-quartz, β-cristobalite and $P6_3/mmc$-tridymite, respectively. The volumes are referenced with respect to a fit to experimental data of the respective structure. The numbers over the bars indicate the relative difference in percent.

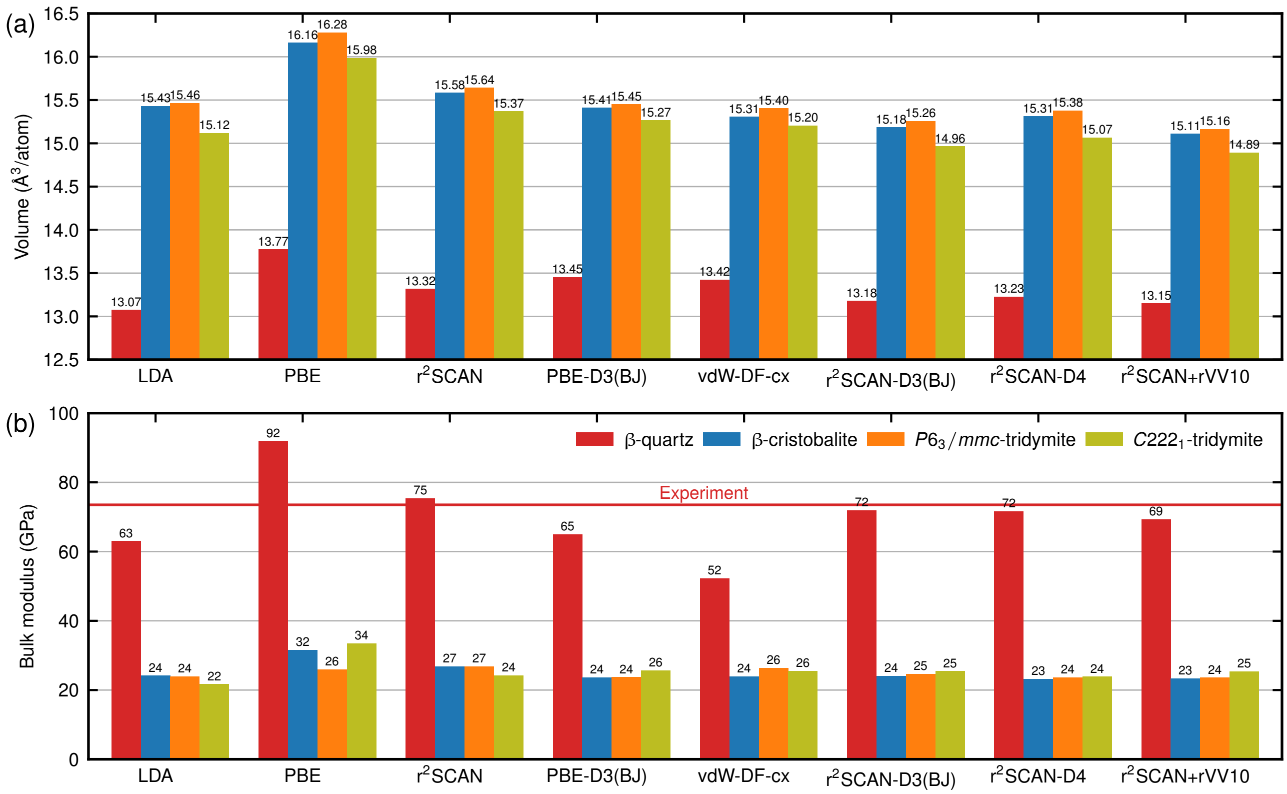

FIG. S3. Rung-1–3 absolute (a) volume and (b) adiabatic bulk modulus at 1137 K of β-quartz, β-cristobalite and $P6_3/mmc$- and $C222_1$-tridymite. The red line marks the experimental bulk modulus for quartz [5, 11].

tive Helmholtz energy differences (1137 K, volume fixed to 13.2/15.2 Å^3/atom for β-quartz/β-cristobalite) for the functionals of rungs 1–3 (corresponding to Fig. 4 in the main text) are repeated in Fig. S6(b). The correlation is very strong in both cases, resulting in a slope of 0.074 k_B. The R^2 value is slightly better for the Gibbs energy, corresponding to a standard deviation of the fit of 0.1 meV/atom or 16 K, as compared to 0.35 meV/atom or 55 K for the Helmholtz energy. The correlation shown in Fig. S6(b), jointly with the free-energy perturbation in the exchange-correlation space, is the basis of the efficient $T_{\text{q-c}}$ prediction for higher-rung functionals in the proposed approach. Predicted $T_{\text{q-c}}$'s are given in Table S2.

The linearity of the $\Delta G(T)$'s between β-cristobalite and β-quartz also enables an efficient and numerically stable prediction of $\Delta S_{\text{q-c}}$. Specifically, a linear correction to the slopes of the Helmholtz energy with respect to temperature is computed [see Eq. (2) in the main text]. For HSE06 and HSE06-D4, the Helmholtz energy difference was computed at three temperature points (1135, 1419 and 1702 K) to validate the assumption of linearity. The correction is indeed very linear as seen in Fig. S7. For RPA, two points were used, at 1135 and 1419 K. The resulting transition entropies are given in Table S2.

D. Equilibrium volume estimates

The equilibrium volume of a certain phase at a given temperature can be estimated for a higher-rung functional by upsampling from a rung-1–3 functional at two volume points. This is possible due to the linear (in fact, nearly constant) volume dependence of the upsampling term. Utilizing the r^{2}SCAN MTP as the reference, we have estimated the equilibrium volumes for β-quartz and β-cristobalite for the rung-4 hybrid HSE06 and HSE06-D4 functionals at 1137 K. The upsampling was performed at 13.2 and 14 Å^3/atom, and 15.2 and 16 Å^3/atom, for β-quartz and β-cristobalite, respectively. The estimated equilibrium volumes are given in Table S3. These equilibrium volumes and the impact of the dispersion correction are consistent with the rung-1–3 functionals, in particular with PBE/PBE-D3(BJ), for β-quartz and β-cristobalite.

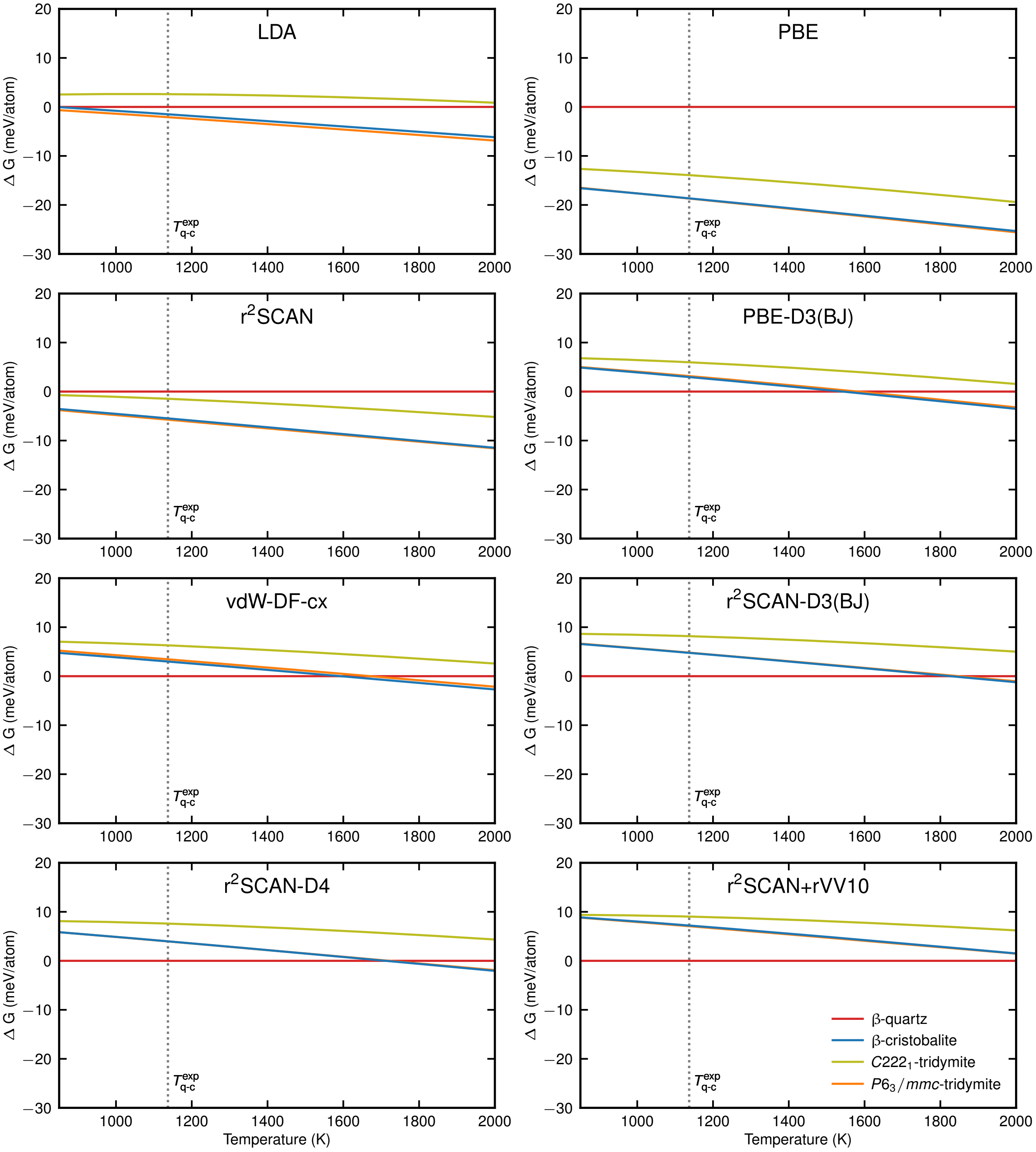

FIG. S4. Gibbs energy difference $\Delta G(T)$ of β-cristobalite and $C222_1$- and $P6_3/mmc$-tridymite with respect to β-quartz at ambient pressure for the functionals in rungs 1–3. If not visible, the orange line is below the blue one. The vertical dashed line marks the CALPHAD transition temperature $T_{\text{q-c}}^{\text{exp}} = 1137\,\text{K}$.

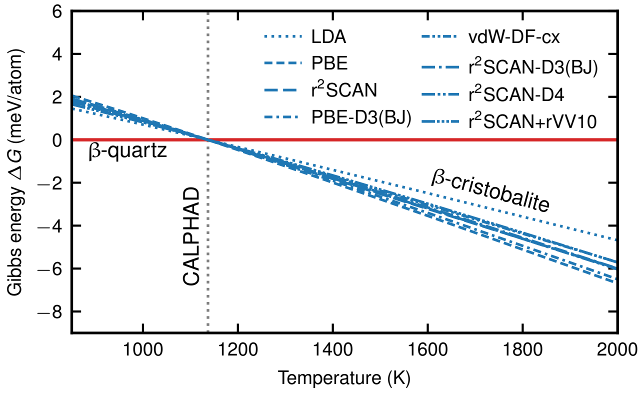

FIG. S5. Shifted Gibbs energy of β-cristobalite with respect to β-quartz, at ambient pressure. The curves have been shifted to be aligned at the CALPHAD transition temperature of 1137 K.

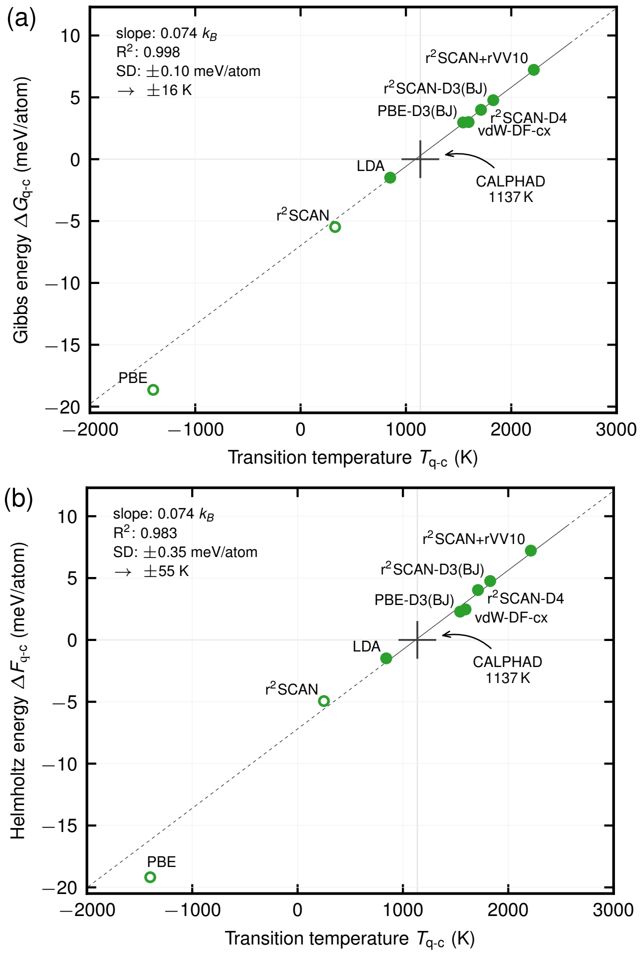

FIG. S6. Correlation between the predicted transition temperature and the (a) Gibbs energy difference $\Delta G_{\text{q-c}}$ and (b) Helmholtz energy difference between β-quartz and β-cristobalite at $T_{\text{q-c}}^{\text{exp}}$, in (b) with fixed volumes. The filled dark green circles show explicitly computed results and the line a linear fit of them. The hollow circles mark extrapolations out of the stability regime of the phases. (b) is a repetition for rungs 1–3 from Fig. 4 in the main text.

TABLE S1. Rung 1–3 transition properties.

	$T_{\text{q-c}}$ (K)	Deviation (%)	$\Delta G_{\text{q-c}}$ (meV/atom)	$\Delta S_{\text{q-c}}$ (k_B)
LDA	886	−22	−1.21	0.052
PBE[a]	−1399	−223	−18.65	0.085
r²SCAN[a]	243	−79	−5.36	0.067
PBE-D3(BJ)	1545	36	2.98	0.088
vdW-DF-cx	1595	40	3.00	0.077
r²SCAN-D3(BJ)	1843	62	4.90	0.083
r²SCAN-D4	1731	52	4.06	0.081
r²SCAN+rVV10	2229	96	7.20	0.082
CALPHAD Average	1137	0	0.00	0.118

[a] The values corresponding to these functionals are extrapolated from the dynamically stabilized regime.

TABLE S2. Rung 4 and 5 transition properties.

	$T_{\text{q-c}}$ (K)	Deviation (%)	$\Delta F_{\text{q-c}}$ (meV/atom)	$\Delta S_{\text{q-c}}$ (k_B)
HSE06[a]	−1723	−252	−18.25	0.060
HSE06-D4	1945	71	5.30	0.084
RPA	1081	−5	−0.26	0.088
CALPHAD Average	1137	0	0.00	0.118

[a] The value corresponding to HSE06 is extrapolated.

TABLE S3. Rung 4 equilibrium volumes (Å³/atom) at $T_{\text{q-c}}^{\text{exp}}$.

	β-quartz	β-cristobalite
HSE06	13.48	16.35
HSE06-D4	13.22	15.29

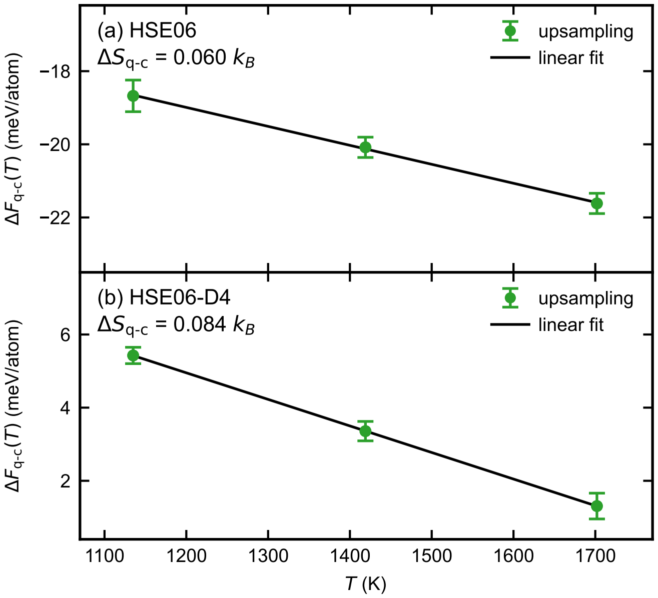

FIG. S7. Calculation of the transition entropy, $\Delta S_{\text{q-c}}$, from the slopes of the Helmholtz energy, $\Delta F_{\text{q-c}}$, for (a) HSE06 and (b) HSE06-D4. The error bars denote statistical errors within a 95% confidence interval.

S3. METHODS

A condensed overview reflecting the computational complexity of the different steps/methods used in this work can be found in Table S4. For a detailed step-by-step description and analysis of the direct-upsampling method, see Refs. [17, 18]. In the following sections, we present only the relevant methodological details and parameters used in the present work, as well as specific convergence tests and RPA parameters. In addition, we also supply the expansion for free-energy perturbation.

A. Detailed computational parameters

As stated in the main text, the direct upsampling was performed in the extended version of Ref. [18], due to the dynamical instability at 0 K of the investigated phases. In particular, effective equations of state were used as the basis for the finite-temperature calculations. These equations of state were adjusted to allow for reasonable thermodynamic integration. Further, an effective harmonic reference fitted to high temperatures was used as a reference for the thermodynamic integration. The supercell sizes for the different steps of the direct upsampling method are summarized in Table S5.

For the thermodynamic integration for each phase, one moment-tensor potential [19] (MTP) was trained [20]. In Table S6, the parameters for the low-DFT molecular dynamics (MD) runs for the initial low-MTP training are given. Table S7 contains parameters for the snapshot generation with the low-MTP. The parameters for the low and high-MTP are given in Tables S8 and Table S9, respectively. The training energy and force root-mean-square errors (RMSEs) of different levels for the high-MTP are shown in Fig. S8(a) and (b), respectively. The corresponding RMSEs for a test set (of equal number of atoms to the training set) are shown in Fig. S8(c) and (d).

The parameters for the effective harmonic potentials fitted to high-temperature snapshots calculated with high-DFT are compiled in Table S10. Effective *harmonic* potentials were used instead of quasiharmonic ones because of a non-trivial volume dependence of the anharmonic contribution, mainly due to soft modes corresponding to the rotation of SiO_4 tetrahedra. Further, effective harmonic potentials were only fitted for LDA and then used as a reference for all functionals.

The parameters for the high-DFT calculations of the snapshots are given in Table S11. The same DFT settings apply to the upsampling. The utilized k-point densities of 192 to 288 k points $\cdot$ atoms (kp $\cdot$ atoms) are sufficient for high convergence due to the insulating character of SiO_2, as explicitly confirmed (see Sec. S3 B). This contrasts with metallic systems, where at least an order of magnitude denser k-point samplings are required (e.g., Ref. [18]). Regarding the occupation function, all calculations were performed by applying the Gaussian smearing with a width of 0.1 eV.

Thermodynamic-integration and upsampling grids and supercell sizes can be seen in Table S12. The anharmonic Helmholtz energy F^{ah}—explicitly available at specific V and T points from the previous steps—was parametrized in V and T with the basis sets shown in Table S13.

For β-quartz, a fixed c/a lattice-parameter ratio was used for all simulations. It was fixed to 1.1, which is close to the experimental value [21]. In addition, careful tests were performed at c/a-ratios of 1.09 and 1.08, and the effect on Helmholtz energy was below 0.5 meV/atom even for these relatively large changes of the ratio. In experiments and calculations with LDA and GGA for α-quartz and idealized β-quartz at 0 K, the changes in c/a ratio were found to be much smaller [10]. These small changes are expected to have a negligible impact on the Helmholtz energy. For $P6_3/mmc$-tridymite, a fixed c/a ratio of 1.64 was used.

TABLE S4. Methodological overview. Steps 1 and 2 are part of the "standard" direct-upsampling method, including both thermodynamic integration (TI) and free-energy perturbation (FEP). Steps 3 and 4 are new developments in this work. Details regarding the fourth step are given in Sec. S3 C.

Step	Method	Level	Supercell	No. atoms	No. V,T points	No. MD steps/snapshots	Plane-wave cutoff (eV)
1	TI	MTP	Large-size	4608–6480	100	100 000	—
2	FEP	rungs 1–3	Medium-size	192–288	100	100	600
3	FEP	rungs 4, 5	Medium-size	192–288	1	100	600, 500
4	FEP	rung 5	Unit cell	9–24	1	1	1000

TABLE S5. Supercells used in the direct-upsampling method. Here $\Delta F^{\mathrm{qh}\to\mathrm{MTP}}$ denotes the difference in Helmholtz energy with respect to the effective *harmonic* potential.

	Small-size		Medium-size		Large-size	
	Supercell	Atoms	Supercell	Atoms	Supercell	Atoms
β-cristobalite	$1\times1\times1$	24	$2\times2\times2$	192	$6\times6\times6$	5184
β-quartz	$2\times2\times2$	72	$3\times3\times3$	243	$8\times8\times8$	4608
$P6_3/mmc$-tridymite	$2\times2\times1$	48	$3\times3\times2$	216	$9\times9\times6$	5832
$C222_1$-tridymite	–	–	$3\times2\times2$	288	$9\times5\times6$	6480
Used for:	Low-MTP fitting		High-MTP fitting, direct upsampling ΔF^{up}		Thermodynamic integration $\Delta F^{\mathrm{qh}\to\mathrm{MTP}}$	

TABLE S6. Low-converged DFT (low-DFT) parameters for *ab initio* MD at high temperatures. The low-DFT runs for the low-MTP fitting were performed with LDA. The LDA-based low-MTPs were used for the fitting of all high-MTPs.

	Input								Output
	Supercell	Atoms	Cutoff[a] (eV)	k points	Volume (AA^3/atom)	Time step (fs)	Steps	Temperature (K)	Min. distance[c] (Å)
β-cristobalite	$1\times1\times1$	24	400	$2\times2\times2$	14.29 ... 16.88, 4 V's	5	1000	1986[b]	0.51
β-quartz	$2\times2\times2$	72	400	$2\times2\times2$	12.02 ... 14.39, 4 V's	5	1000	1986[b]	1.30
$P6_3/mmc$-tridymite	$2\times2\times1$	48	400	$2\times2\times2$	14.73 ... 17.54, 16 V's	5	1000	1500	1.29

[a] The energy cutoff corresponds to the maximum `ENMAX`.
[b] This is the experimental melting temperature.
[c] "Min. distance" is the minimum distance between any two atoms during the MD.

TABLE S7. Snapshot-generation parameters with low-MTP MD in medium-size supercells at the experimental melting point of 1986 K.

	Input					Output	
	Supercell	Atoms	Volume (Å^3/atom)	Temperature (K)	Initial snapshots[a]	Selected snapshots[a]	Min. distance (Å)
β-cristobalite							
LDA	$2\times2\times2$	192	14.01 ... 15.55, 4 V's × 3 c/a's	200, 400, 1986	2660	490	1.38
PBE-D3(BJ)	$2\times2\times2$	192	14.0 ... 16.0, 8 V's	1986	240	236	1.37
r^{2}SCAN	$2\times2\times2$	192	14.0 ... 16.0, 8 V's	1986	231	171	1.38
β-quartz							
LDA	$3\times3\times3$	243	12.02 ... 14.39, 8 V's	1986	240	204	1.39
PBE-D3(BJ)	$3\times3\times3$	243	12.0 ... 14.0, 8 V's	1986	240	188	1.38
r^{2}SCAN	$3\times3\times3$	243	12.0 ... 14.0, 8 V's	1986	240	196	1.37
$P6_3/mmc$-tridymite							
LDA	$3\times3\times2$	216	14.73 ... 17.54, 8 V's	1500	240	164	1.41
PBE-D3(BJ)	$3\times3\times2$	216	14.73 ... 17.54, 8 V's	1500	240	164	1.39
r^{2}SCAN	$3\times3\times2$	216	14.73 ... 17.54, 8 V's	1500	240	162	1.38

[a] Initial snapshots are uncorrelated snapshots with a fixed time interval in between, and the selected snapshots are those selected for the training with an extrapolation grade threshold.

TABLE S8. Low-MTP fitting parameters and results. The low-DFT runs for the low-MTP fitting were performed with LDA. The LDA-based low-MTPs were used for the fitting of all high-MTPs.

	Input				Output		
	R_{min} (Å)	R_{cut} (Å)	Level	No. of MTP parameters	Energy RMSE (meV/atom)	Force RMSE (eV/Å)	ΔF^{up} (meV/atom)
β-cristobalite	2.0	6.0	12	128	5.4	1.686	–
β-quartz	1.30	6.0	10	115	2.7	0.165	–
$P6_3/mmc$-tridymite	1.28	6.0	10	115	2.2	0.114	–

TABLE S9. High-MTP fitting parameters and results. The minimum distance and cutoff radius of the MTP are denoted as R_{min} and R_{cut}, respectively. The ΔF^{up} column refers to the Helmholtz energy difference between the MTP and the respective exchange-correlation functional. This difference is evaluated at the (V,T) grid point closest to $T^{\mathrm{exp}}_{\text{q-c}}$ and the corresponding volume.

	Input				Output		
	R_{min} (Å)	R_{cut} (Å)	Level	No. of MTP parameters	Energy RMSE (meV/atom)	Force RMSE (eV/Å)	ΔF^{up} (meV/atom)
β-cristobalite							
LDA	2.0	6.0	16	223	0.52	0.066	0.07
PBE-D3(BJ)	1.35	6.0	16	223	0.97	0.087	0.18
r^2SCAN	1.37	6.0	16	223	1.06	0.093	0.12
β-quartz							
LDA	1.37	6.0	18	326	0.73	0.091	0.09
PBE-D3(BJ)	1.38	6.0	16	223	1.06	0.109	0.10
r^2SCAN	1.36	6.0	18	326	1.08	0.108	0.11
$P6_3/mmc$-tridymite							
LDA	1.39	6.0	16	223	0.60	0.086	0.13
PBE-D3(BJ)	1.38	6.0	16	223	1.02	0.097	0.13
r^2SCAN	1.38	6.0	16	223	1.12	0.104	0.12

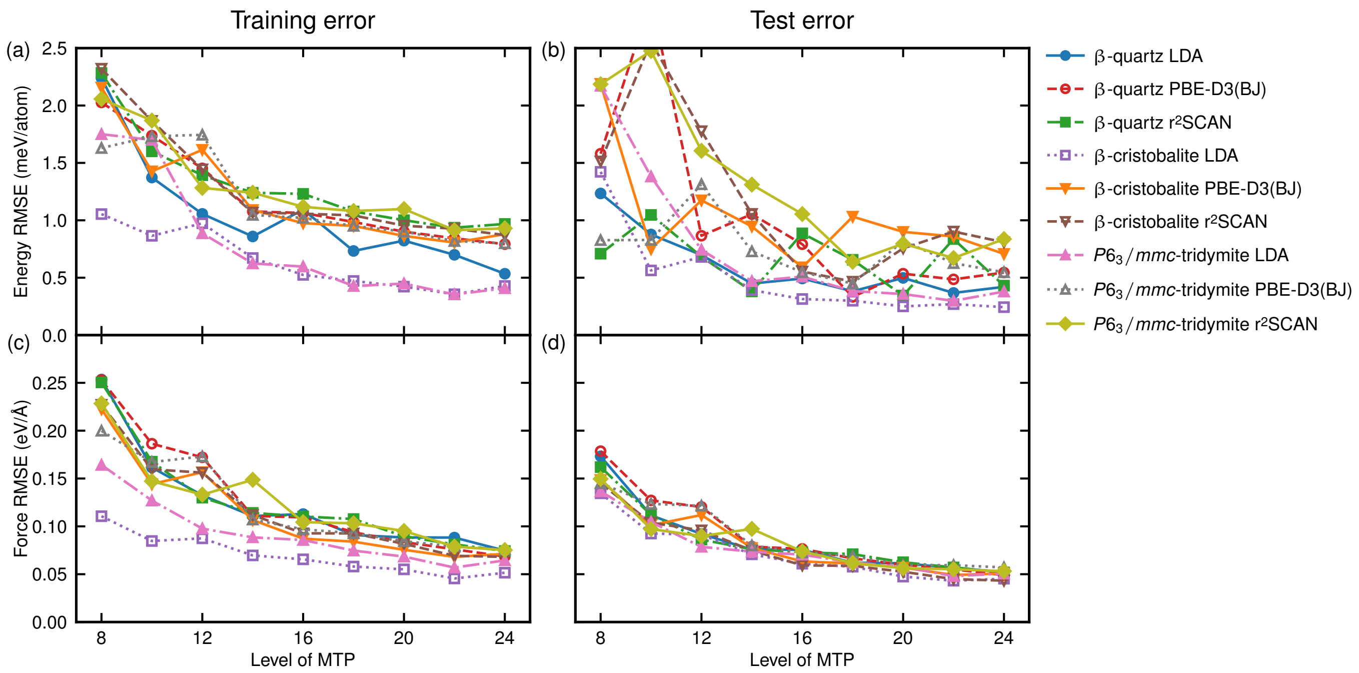

FIG. S8. Training and test errors of the high-MTPs. The training root-mean-squared errors (RMSEs) of the (a) energy per atom and (c) force components. The MTPs are trained in medium-size supercells to LDA, PBE-D3(BJ), and r^2SCAN DFT energies and forces of configurations at high temperatures in the relevant V range. Test RMSEs of the (b) energy per atom and (d) force components corresponding to configurations of the upsampling at the T grid point of transition from β-quartz to β-cristobalite $T^{\mathrm{exp}}_{\text{q-c}} = 1137$ K and the equilibrium V.

TABLE S10. Parameters for fitting effective harmonic potentials to high-DFT forces from high-MTP MD snapshots. Only LDA high-MTPs were used, and LDA harmonic potentials were fitted. The latter were then used for all functionals.

	Input								Output	
	Fitting		Extended dyn. mat.							
	Supercell	Atoms	Supercell	Atoms	Volume (Å^3/atom)	Temperature (K)	R_{cut} (Å)	N_{param}[a]	R^2	Force RMSE (eV/Å)
β-cristobalite	$2\times2\times2$	192	$6\times6\times6$	5184	15.71	1500	6.6	60	0.72	1.09
β-quartz	$3\times3\times3$	243	$8\times8\times8$	4608	13.57	1000	6.5	312	0.86	0.61
$P6_3/mmc$-tridymite	$3\times3\times2$	216	$9\times9\times6$	5832	16.09	1500	6.6	206	0.77	0.98
$C222_1$-tridymite [b]	$3\times2\times2$	288	$9\times5\times6$	6480	16.00	1986	4.5	295	0.71	1.23

[a] N_{param} is the number of fitting parameters.
[b] The $C222_1$-tridymite effective harmonic potential was fitted to PBE-D3(BJ).

TABLE S11. High-DFT parameters in medium-size supercells. The settings are the same for all rung 1–3 functionals.

	Supercell	Atoms	Cutoff[a] (eV)	k points	kp · atoms
β-cristobalite	$2\times2\times2$	192	600	$1\times1\times1$	192
β-quartz	$3\times3\times3$	243	600	$1\times1\times1$	243
$P6_3/mmc$-tridymite	$3\times3\times2$	216	600	$1\times1\times1$	216
$C222_1$-tridymite	$3\times2\times2$	288	600	$1\times1\times1$	288

[a] The energy cutoff corresponds to the maximum $1.5\times$ ENMAX in the VASP POTCAR.

TABLE S12. Parameters for the anharmonic calculations; thermodynamic integration (TI) from effective harmonic potential to high-MTP and upsampling (or FEP) from high-MTP to high-DFT. For the other functionals in rungs 1–3 only upsampling was performed from PBE-D3(BJ) and r^{2}SCAN with the same cells and on the same grids as listed here.

	TI		Upsampling/FEP				
	Supercell	Atoms	Supercell	Atoms	Snapshots[a]	Volume (Å^3/atom)	Temperature (K)
β-cristobalite							
LDA	$6\times6\times6$	5184	$2\times2\times2$	192	80	14.21 ... 15.93, 25 V's	1986/7×{3, 4, 5, 6, 7, 7.5, 8, 9, 10}
PBE-D3(BJ)	$6\times6\times6$	5184	$2\times2\times2$	192	80	14.00 ... 16.00, 11 V's	1986/7×{3, 4, 5, 6, 7, 7.5, 8, 9}
r^{2}SCAN	$6\times6\times6$	5184	$2\times2\times2$	192	80	14.00 ... 16.00, 11 V's	1986/7×{3, 4, 5, 6, 7, 7.5, 8, 9}
β-quartz							
LDA	$8\times8\times8$	4608	$3\times3\times3$	243	80	12.21 ... 14.39, 12 V's	1986/7×{3, 4, 5, 6, 7, 7.5, 8, 9, 10}
PBE-D3(BJ)	$8\times8\times8$	4608	$3\times3\times3$	243	80	13.00 ... 14.00, 11 V's	1986/7×{3, 4, 5, 6, 7, 7.5, 8, 9}
r^{2}SCAN	$8\times8\times8$	4608	$3\times3\times3$	243	80	12.00 ... 14.00, 11 V's	1986/7×{3, 4, 5, 6, 7, 7.5, 8, 9}
$P6_3/mmc$-tridymite							
LDA	$9\times9\times6$	5832	$3\times3\times2$	216	80	14.15 ... 16.09, 20 V's	1986/7×{3, 4, 5, 6, 7, 7.5, 8, 9, 10}
PBE-D3(BJ)	$9\times9\times6$	5832	$3\times3\times2$	216	80	14.00 ... 16.00, 11 V's	1986/7×{3, 4, 5, 6, 7, 7.5, 8, 9}
r^{2}SCAN	$9\times9\times6$	5832	$3\times3\times2$	216	80	14.00 ... 16.00, 11 V's	1986/7×{3, 4, 5, 6, 7, 7.5, 8, 9}
$C222_1$-tridymite							
LDA	$9\times5\times6$	6480	$3\times2\times2$	288	80	14.00 ... 16.00, 11 V's	1986/7×{3, 4, 5, 6, 7, 7.5, 8, 9}
PBE-D3(BJ)	$9\times5\times6$	6480	$3\times2\times2$	288	80	14.00 ... 16.00, 11 V's	1986/7×{3, 4, 5, 6, 7, 7.5, 8, 9}
r^{2}SCAN	$9\times5\times6$	6480	$3\times2\times2$	288	80	14.00 ... 16.00, 11 V's	1986/7×{3, 4, 5, 6, 7, 7.5, 8, 9}

[a] The high-DFT sampling of snapshots is finished when the estimated deviation becomes less than a target value at each (V,T) grid point. The number of sampled snapshots is variable, and the maximum numbers of snapshots are listed.

TABLE S13. Basis-set elements used for the parametrization of the anharmonic Helmholtz energy contribution F^{ah}.

Order	0	1	2	3	4
Basis element[a]	1	V,T	V^2,VT,T^2	V^3,V^2T,VT^2	V^3T,V^2T^2

[a] For β-quartz with the vdW-DF-cx functional, the V^3T-term was excluded.

B. Systematic tests for convergence and accuracy

In Fig. S9, we show convergence tests for cristobalite for the supercell size. The behavior is similar also for tridymite, and for production runs a size of around 5000 atoms was chosen for all phases.

In Fig. S10, we show the convergence with respect to the plane-wave cutoff for the upsampling to r^{2}SCAN. In (a) and (b), we show the energies of five randomly chosen snapshots for β-quartz and β-cristobalite, respectively. By showing the energy offset of each snapshot from the average energy (at each energy cutoff), the effect on second- and higher-order terms in the free-energy perturbation is isolated. (The dependence of the absolute energies on the cutoff is, of course, much more severe.) In (c), we in turn show the dependence of the energy difference between β-quartz and β-cristobalite, calculated from their average snapshot energies. The energy difference between the phases converges slightly slower than the relative energies of the snapshots within each phase. Together, these results ensure that our final values are converged both in terms of upsampling for each phase and in terms of phase stability. Further convergence tests for the k-point density indicate that already around 200 kp · atoms provides very well converged results. Tests conducted up to around 25 000 kp · atoms for upsampling to r^{2}SCAN, show maximum energy differences of only 0.04 meV/atom.

Convergence of the order of the polynomials used for fitting and the (V,T) grid can be seen in Fig. S11 and Fig. S12, respectively. Figure S11 shows the volume dependence of the Helmholtz energy at the β-quartz–β-cristobalite transition temperature and the temperature dependence of the volume and bulk modulus at ambient pressure, for different polynomial basis sets. The 2nd-order basis is not enough to capture the volume dependence of the Helmholtz energy surface. A 3rd-order polynomial basis, on the other hand, captures the temperature dependence well, and only a small change is seen compared to the 4th-order basis in Helmholtz energy and equilibrium volume. For the (V,T)-grid convergence, at least the 11×8 grid is necessary for a sufficiently converged Helmholtz energy. For the bulk modulus, there is a clear difference between 3rd and 4th order polynomial basis, especially for β-quartz. For the bulk modulus of β-quartz there is also a higher sensitivity to the (V,T)-grid density. Since the main focus of this work is phase stabilities and transition properties, we have used the 4th-order basis as presented in Table S13 and at least an 11×8 (V,T) grid for the production calculations.

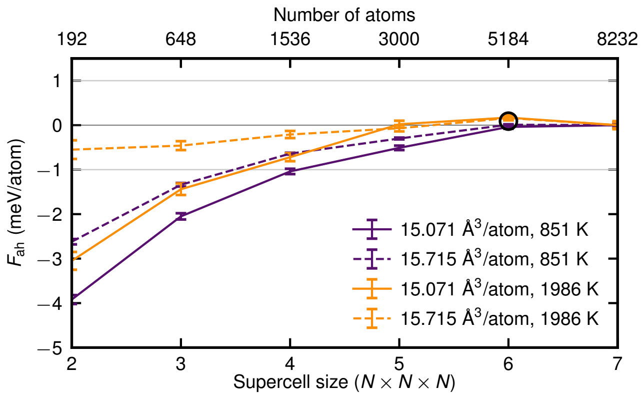

FIG. S9. Convergence of the anharmonic Helmholtz energy F^{ah} with respect to supercell size for β-cristobalite computed with the LDA MTP. The error bars indicate the statistical errors within a 95% confidence interval, and the black circle marks the size used for TI in the production runs.

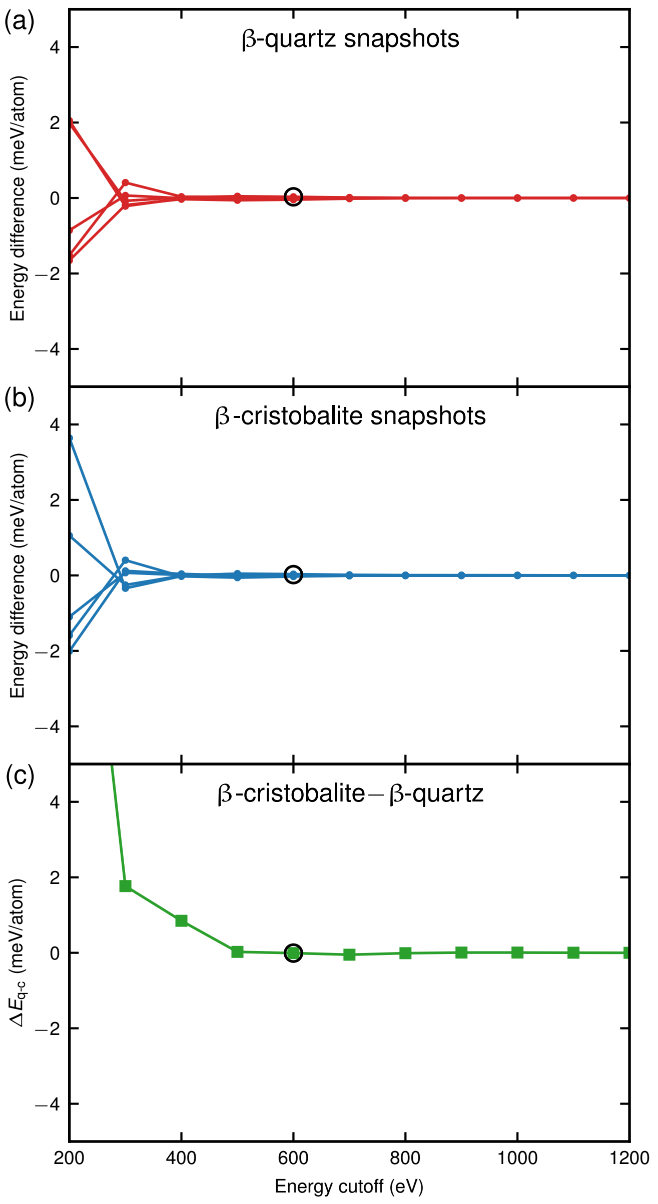

FIG. S10. r^{2}SCAN energy convergence with respect to the plane-wave cutoff (`ENCUT`), between snapshots within the same phase [(a) and (b)] and for the snapshot-average difference between β-quartz and β-cristobalite $\Delta E_{\text{q-c}}$ in (c). In (a) and (b), the energies are referenced with respect to the average snapshot energy for each cutoff. All curves are shifted with respect to their last value. The values used for production calculations are marked by a circle.

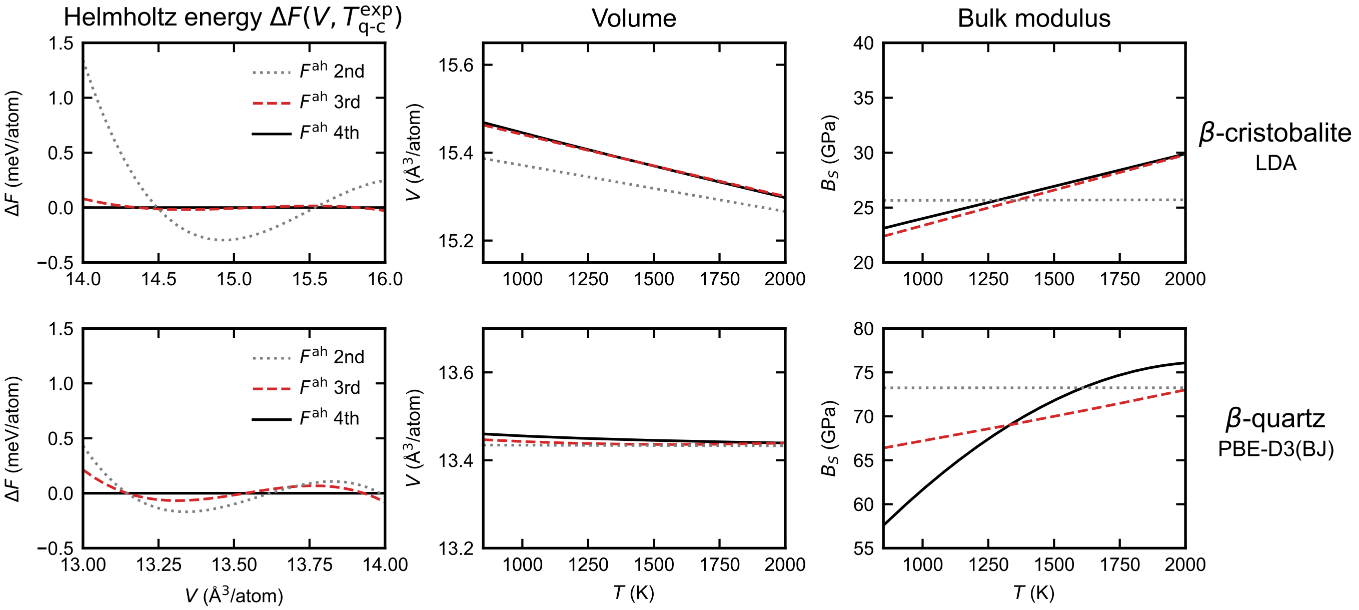

FIG. S11. Dependence of thermodynamic properties on the order of the polynomial basis used in the parametrization of the anharmonic Helmholtz energy $F^{\mathrm{ah}}(V,T)$, for β-cristobalite with LDA and β-quartz with PBE-D3(BJ), sampled on a $11V \times 8T$ grid. Here, $\Delta F(V, T_{\text{q-c}}^{\text{exp}})$ denotes the difference in Helmholtz energy from the polynomial with the highest order 4, at 1137 K. In addition, the volume and the adiabatic bulk modulus B_S are shown.

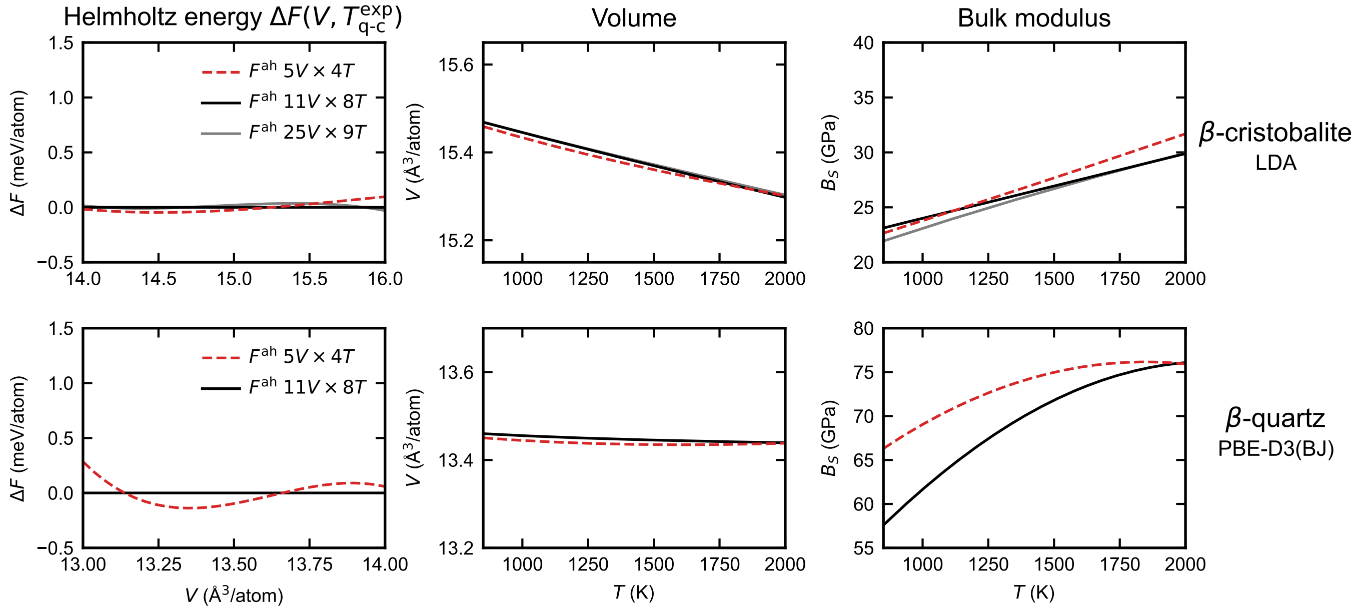

FIG. S12. Dependence of thermodynamic properties on the grid density for the anharmonic Helmholtz energy $F^{\mathrm{ah}}(V,T)$. Here, $\Delta F(V, T_{\text{q-c}}^{\text{exp}})$ denotes the difference in Helmholtz energy from the $11V \times 8T$ grid at 1137 K, and B_S the adiabatic bulk modulus.

C. RPA details and convergence tests

A special procedure to obtain the upsampled RPA Helmholtz energy is described in the Methods section in the main text. The final RPA Helmholtz energy is in the end obtained using Eq. (5). The intermediate energies for the steps in the procedure are detailed in Table S14, Table S15, and Table S16.

In order to guarantee the validity and precision of the approach, extensive convergence tests were performed. In Fig. S13, we show the dependence of absolute energies on the number of time/frequency points, at an offset from the highest value. There is, in principle, no dependence on the number of time/frequency points, even on the absolute scale. Only for higher cutoffs, above 1000 eV, could we notice a small time/frequency dependence. The convergence results for these high cutoffs were obtained with an increased number of points, 16 points. The k-point dependence is also weak, on the relative scale. In Fig. S14, we show the dependence of the energy difference between two snapshots within each phase, respectively, as well as the energy difference between β-quartz and β-cristobalite. This applies both to the RPA correlation energy and the exact exchange energy when a cutoff for the Coulomb kernel is used for the Fock and exchange energy evaluation. (Here, we used HFRCUT $= -1$ for an automated cutoff in VASP.) Still, we applied the first-order correction to the exact exchange energy and finally used 1125/1536 kp $\cdot$ atoms for β-quartz/β-cristobalite. For $\Delta F^{\mathrm{up}}_{\mathrm{normal}}$ only the Γ point was sampled, i.e., 243/192 kp $\cdot$ atoms for β-quartz/β-cristobalite.

The energy-cutoff convergence is more severe, as seen in Fig. S15. In the left column, the convergence of the exact exchange energy is shown, and in the right column, that of the RPA correlation energy. In a similar way as in Fig. S10, we show the convergence of β-quartz snapshots in (a) and (b), the convergence of β-cristobalite snapshots in (c) and (d), and finally the energy difference between β-quartz and β-cristobalite in (e) and (f). Additionally, a thicker line is shown in (a)–(d), indicating the convergence of the small snapshot with respect to the snapshot average. Using the converged cutoff marked by circles guarantees that the first-order correction is valid and yields precise results. The phase energy difference in (e) and (f) was calculated with the small snapshot for each phase. Using the small snapshot, we could also investigate the effect of the Si core polarization—in practice by performing RPA calculations with a PAW potential including the Si $2s$ and $2p$ electrons in the valence shell.

In Table S17, the RPA computational parameters are presented. For smooth convergence and accurate results, `PRECFOCK=Normal` for both exact exchange and RPA calculations was important. `PRECFOCK=Accurate` gave only very small differences in energy in the investigated system. The computational load increased by around 25% when using `PRECFOCK=Normal` compared to `PRECFOCK=Fast`. On the other hand, using `PRECFOCK=Normal` allowed for using at least 50 eV lower `ENCUT` (500 eV instead of 550 eV), thereby almost fully compensating for using `PRECFOCK=Normal` in terms of computation time. Additionally, `PRECFOCK=Normal` was important to obtain an agreement between the energies per atom of the scaled and the small cells, when computed with corresponding settings. For the calculations of the exact exchange energy, `HFRCUT` was important for a reasonable k-point convergence. For the correlation calculations, PBE orbitals gave the same result as LDA orbitals (considering the same set of snapshots), and we used the former ones.

D. Expansion of free-energy perturbation

The convergence behavior of the upsampling is quantified by the second-order term of the expansion of the thermodynamic free-energy perturbation [23] formula in Eq. (4) in the main text:

$$\Delta F^{\mathrm{up}}(V,T) = -k_B T \ln \left\langle \exp\left(-\frac{\Delta E}{k_B T}\right)\right\rangle_{\mathrm{ML}} = \omega_1 - \omega_2/2k_BT + O\left((1/k_BT)^2\right), \tag{S6}$$

where $\omega_1 = \langle \Delta E\rangle_{\mathrm{ML}}$, $\omega_2 = \left\langle(\Delta E)^2\right\rangle_{\mathrm{ML}} - \langle\Delta E\rangle^2_{\mathrm{ML}}$, and $\Delta E = E - E^{\mathrm{ML}}$, with the energies E and E^{ML} calculated with the functional of interest and the ML potential, respectively. The second term of the second line of Eq. (S6),

$$e_2 = -\frac{\omega_2}{2k_BT} = -\frac{1}{2k_BT}\left[\frac{1}{N_{\mathrm{s}}}\sum_{i=1}^{N_{\mathrm{s}}}\Delta E_i^2 - \left(\frac{1}{N_{\mathrm{s}}}\sum_{i=1}^{N_{\mathrm{s}}}\Delta E_i\right)^2\right], \tag{S7}$$

is called the second-order term. The right-hand side of Eq. (S7) expresses the second-order term in the notation of Sec. S1 A.

TABLE S14. Upsampling results from PBE-D3(BJ) MTP snapshots to RPA with “normal” settings, i.e., a plane-wave energy cutoff of 500 eV. The statistical error of $\Delta F^{\mathrm{up}}_{\mathrm{normal}}$ is given within a 95% confidence interval and is estimated through the delta method [22].

	$\Delta F^{\mathrm{up}}_{\mathrm{normal}}$ (meV/atom)	Second-order term (meV/atom)	Number of snapshots
β-quartz	−16 206.57 ± 0.18	−0.45	51
β-cristobalite	−16 211.23 ± 0.23	−0.62	51

TABLE S15. RPA first-order correction $E_{\mathrm{high}} - E_{\mathrm{normal}}$ (from 500 to 1000 eV plane-wave energy cutoff) and the separate terms E_{normal} and E_{high} for β-quartz and β-cristobalite. The contributions from exchange and correlation are given together with the total value for each phase. The unit is meV/atom.

	E_{normal}	E_{high}	$E_{\mathrm{high}} - E_{\mathrm{normal}}$
β-quartz			
Exchange	−15 979.41	−15 978.94	0.47
Correlation	−8170.63	−8263.35	−92.71
Total	−24 150.05	−24 242.28	−92.24
β-cristobalite			
Exchange	−15 951.44	−15 951.00	0.44
Correlation	−8171.26	−8263.32	−92.05
Total	−24 122.70	−24 214.32	−91.62

TABLE S16. Final RPA results. The statistical error is given within a 95% confidence interval and is estimated through the delta method [22]. The unit is meV/atom.

	β-quartz	β-cristobalite	Difference $\Delta F_{\mathrm{q\text{-}c}}$
$\Delta F^{\mathrm{up}}_{\mathrm{normal}}$	−16 206.57 ± 0.18	−16 211.23 ± 0.23	−4.65 ± 0.29
$E_{\mathrm{high}} - E_{\mathrm{normal}}$	−92.24	−91.62	0.62
Sum ΔF^{up}	−16 298.81	−16 302.84	−4.04
$F^{\mathrm{ML}}(V, T;$ PBE-D3(BJ))	—	—	2.07
Core polarization shift (Si_sv_GW)	—	—	1.71
RPA (without core polarization)	—	—	−1.97
RPA	—	—	−0.26

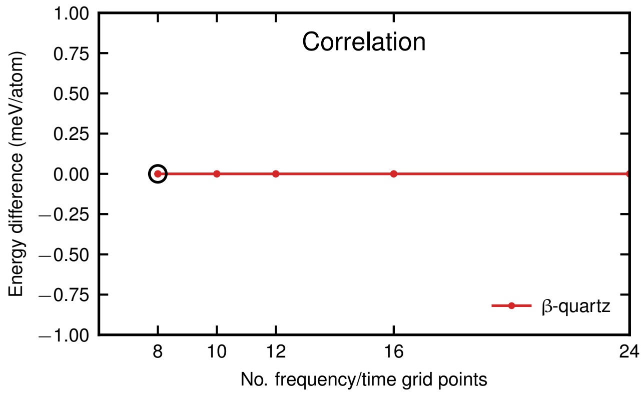

FIG. S13. RPA energy convergence with respect to the number of frequency and time grid points (`NOMEGA`), between two snapshots within β-quartz. The value used for production calculations is marked by a circle. The plane-wave energy cutoff was 450 eV.

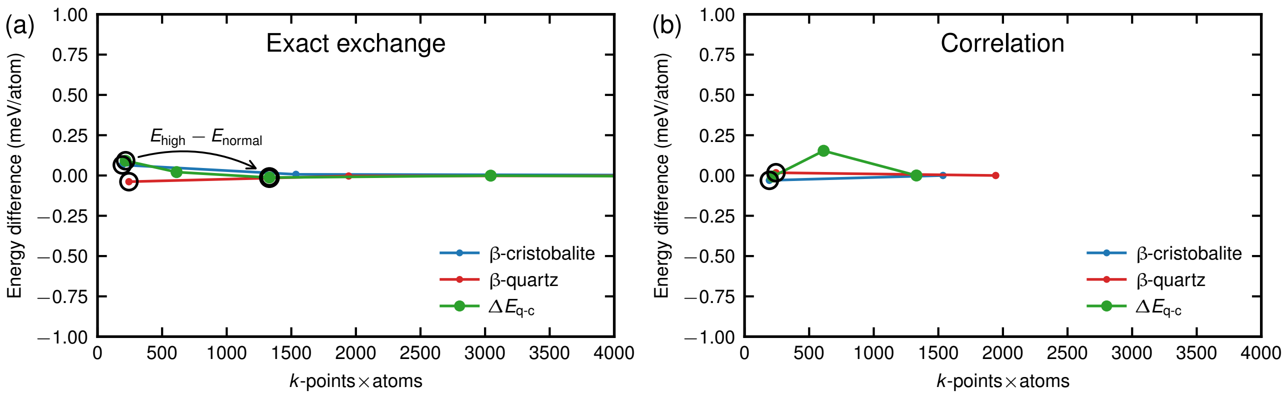

FIG. S14. RPA energy convergence with respect to k points, between two snapshots within the same phase (red and blue) and for the energy difference between one β-quartz and one β-cristobalite snapshot $\Delta E_{\text{q-c}}$ (green), for (a) the exact exchange energy and (b) the correlation energy. The values used for production calculations are marked by a circle. The plane-wave energy cutoff was 400 eV for the exact exchange energy and 350 eV for the correlation, except for the green lines where 650 eV was used. Note that the energy scale differs from Fig. S15 by a factor of 1/5.

TABLE S17. Computational parameters used for the RPA calculations. The “High” settings were used for calculating E_{high} and the “Normal” settings for E_{normal} and $\Delta F^{\text{up}}_{\text{normal}}$.

	Normal convergence (between snapshots)		High convergence (between phases)	
	Exact exchange	Correlation	Exact exchange	Correlation
`PREC`	`Accurate`	`Accurate`	`Accurate`	`Accurate`
`PRECFOCK`[a]	`Normal`	`Normal`	`Normal`	`Normal`
`LMAXFOCKAE`	4	4	4	4
`NOMEGA`	—	8	—	8
`ENCUT`	500	500	1000	1000
`ENCUTGW`	333[b]	333[b]	667[b]	667[b]
`ENCUTGWSOFT`	267[c]	267[c]	533[c]	533[c]
`HFRCUT`	−1	—	−1	—
k-points×atoms β-quartz	243	243	1125[d]	243
k-points×atoms β-cristobalite	192	192	1536[d]	192

[a] `PRECFOCK` affects the FFT grid densities and at least the `Normal` setting was required.
[b] This is the default VASP value (2/3*`ENCUT`).
[c] This is the default VASP value (0.8*`ENCUTGW`).

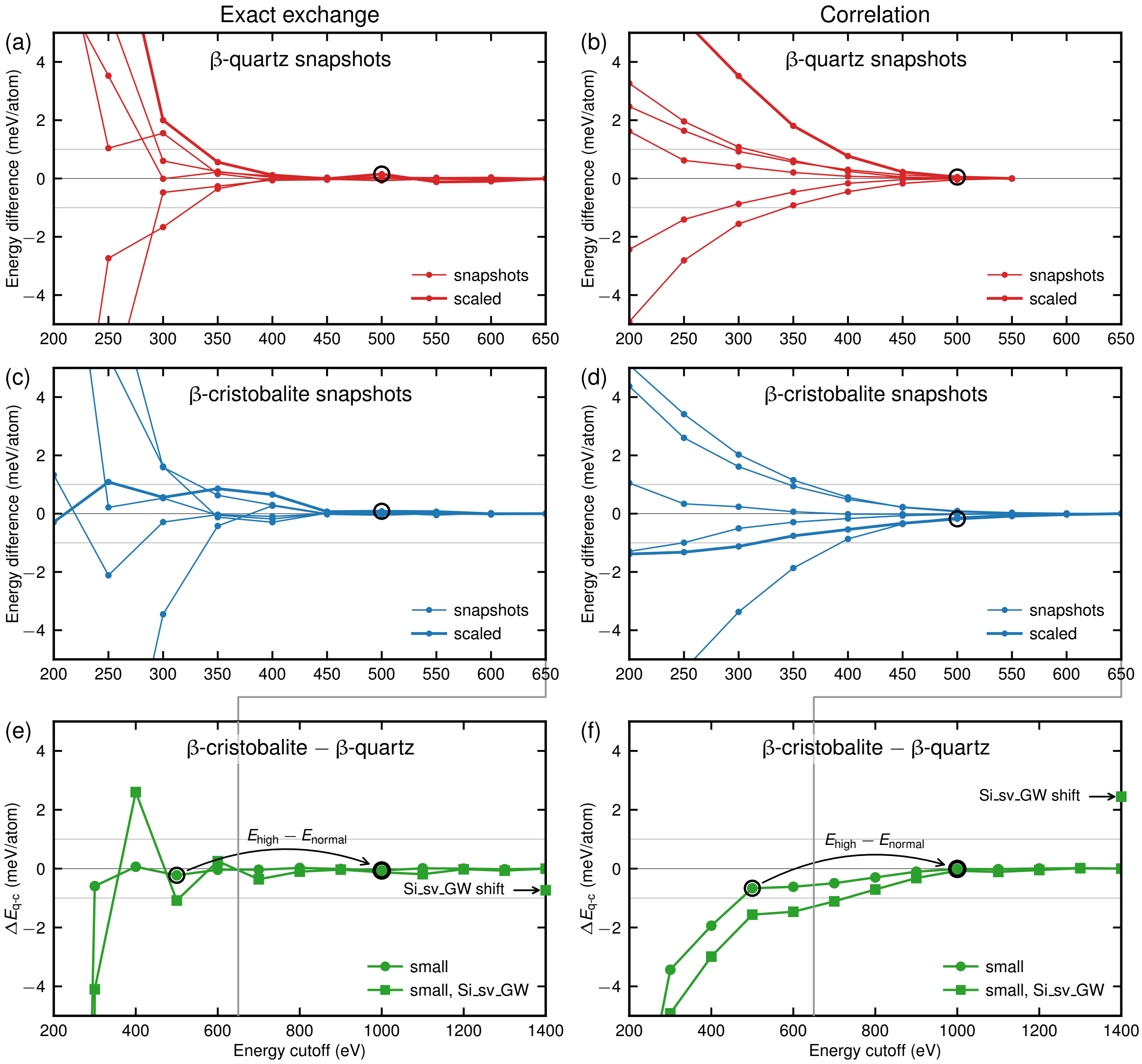

FIG. S15. RPA energy convergence with respect to the plane-wave cutoff (ENCUT), between snapshots within the same phase [(a)–(d)] and for the energy difference between β-quartz and β-cristobalite small cells $\Delta E_{\text{q-c}}$ [(e) and (f)]. In (a)–(d), the energies are referenced with respect to the average snapshot energy for each cutoff. All curves are shifted with respect to their last value. Further, the shifts resulting from core polarization (Si semi-core states included) are marked with additional points. The values used for production calculations are marked by a circle.

* axefor@kth.se

[1] A. Forslund, J. H. Jung, Y. Ikeda, and B. Grabowski, Data for: Free-energy perturbation in the exchange-correlation space accelerated by machine learning: Application to silica polymorphs (2025), DaRUS, V1.

[2] A. H. Larsen, J. J. Mortensen, J. Blomqvist, I. E. Castelli, R. Christensen, M. Dułak, J. Friis, M. N. Groves, B. Hammer, C. Hargus, E. D. Hermes, P. C. Jennings, P. B. Jensen, J. Kermode, J. R. Kitchin, *et al.*, The atomic simulation environment—a Python library for working with atoms, J. Phys.: Condens. Matter **29**, 273002 (2017).

[3] R. J. Ackermann and C. A. Sorrell, Thermal expansion and the high–low transformation in quartz. I. High-temperature X-ray studies, J. Appl. Crystallogr. **7**, 461 (1974).

[4] E. Bourova and P. Richet, Quartz and cristobalite: High-temperature cell parameters and volumes of fusion, Geophys. Res. Lett. **25**, 2333 (1998).

[5] D. L. Lakshtanov, S. V. Sinogeikin, and J. D. Bass, High-temperature phase transitions and elasticity of silica polymorphs, Phys. Chem. Miner. **34**, 11 (2006).

[6] A. F. Wright and A. J. Leadbetter, The structures of the β-cristobalite phases of SiO_2 and $AlPO_4$, Phil. Mag. **31**, 1391 (1975).

[7] I. P. Swainson and M. T. Dove, On the thermal expansion of β-cristobalite, Phys. Chem. Miner. **22**, 61 (1995).

[8] C. Berger, L. Eyrand, M. Richard, and R. Rivière, Etude radiocristallographique de variation de volume pour quelques matériaux subissant des transformation des phases solide-solide, Bull. Soc. Chim. Fr. **2**, 628 (1966).

[9] K. Ohsumi, T. Sawada, Y. Takeuchi, and R. Sadanaga, *Laser-Heating Device for Single-Crystal Diffractometry and Its Application to the Structural Study of High Cristobalite*, Materials science of the Earth's interior (D. Reidel, 1984) pp. 633–643.

[10] T. Demuth, Y. Jeanvoine, J. Hafner, and J. G. Ángyán, Polymorphism in silica studied in the local density and generalized-gradient approximations, J. Condens. Matter Phys. **11**, 3833 (1999).

[11] I. Ohno, K. Harada, and C. Yoshitomi, Temperature variation of elastic constants of quartz across the α - β transition, Phys. Chem. Miner. **33**, 1 (2006).

[12] S. Schnurre, J. Gröbner, and R. Schmid-Fetzer, Thermodynamics and phase stability in the Si–O system, J. Non-Cryst. Solids **336**, 1 (2004).

[13] I. Bajenova, A. Khvan, A. Dinsdale, and A. Kondratiev, Implementation of the extended Einstein and two-state liquid models for thermodynamic description of pure SiO_2 at 1 atm, Calphad **68**, 101716 (2020).

[14] H. Wrledt, The O-Si oxygen-silicon system, Bull. Alloy Phase Diagrams **11**, 43 – 61 (1990).

[15] S. B. Holmquist, Conversion of quartz to tridymite, J. Am. Ceram. Soc. **44**, 82 (1961).

[16] K. Kihara, Thermal change in unit-cell dimensions, and a hexagonal structure of tridymite, Z. Kristallogr. Cryst. Mater. **148**, 237 (1978).

[17] J. H. Jung, P. Srinivasan, A. Forslund, and B. Grabowski, High-accuracy thermodynamic properties to the melting point from ab initio calculations aided by machine-learning potentials, npj Comput. Mater. **9**, 3 (2023).

[18] J. H. Jung, A. Forslund, P. Srinivasan, and B. Grabowski, Dynamically stabilized phases with full *ab initio* accuracy: Thermodynamics of Ti, Zr, Hf with a focus on the hcp-bcc transition, Phys. Rev. B **108**, 184107 (2023).

[19] A. V. Shapeev, Moment Tensor Potentials: A Class of Systematically Improvable Interatomic Potentials, Multiscale Model Simul. **14**, 1153 (2016).

[20] Except in the case of $C222_1$-tridymite, for which the $P6_3/mmc$ MTP was used.

[21] A. Wright and M. Lehmann, The structure of quartz at 25 and 590°C determined by neutron diffraction, Journal of Solid State Chemistry **36**, 371 (1981).

[22] A. Pohorille, C. Jarzynski, and C. Chipot, Good Practices in Free-Energy Calculations, J. Phys. Chem. B **114**, 10235 (2010).

[23] R. W. Zwanzig, High-Temperature Equation of State by a Perturbation Method. I. Nonpolar Gases, J. Chem. Phys. **22**, 1420 (1954).
